# Reaction‐Induced Reversible Reconstruction Enhanced Ni‐MgO/CaO Dual Functional Material for Stable CO_2_ Capture and In Situ Conversion

**DOI:** 10.1002/advs.75455

**Published:** 2026-04-27

**Authors:** Hao Xu, Chen Hou, Jiawei Zhong, Chuande Huang, Wei Wei, Jiawei Hu

**Affiliations:** ^1^ Center For Low‐Carbon Conversion Science and Engineering State Key Laboratory of Low Carbon Catalysis and Carbon Dioxide Utilization Shanghai Advanced Research Institute Chinese Academy of Sciences Shanghai China; ^2^ University of Chinese Academy of Sciences Beijing China; ^3^ Shanghai Synchrotron Radiation Facility Shanghai Advanced Research Institute Chinese Academy of Sciences Shanghai China; ^4^ Institute of Biomass Engineering South China Agricultural University Guangzhou China; ^5^ CAS Key Laboratory of Science and Technology on Applied Catalysis Dalian Institute of Chemical Physics Chinese Academy of Sciences Dalian China

**Keywords:** calcium looping, integrated CO_2_ capture and utilization, metal‐support interaction, methane dry reforming, solid solution

## Abstract

Calcium looping dry reforming of methane (CaLDRM), which realizes CO_2_ capture and in situ conversion to valuable syngas, presents a promising route for large‐scale CO_2_ fixation. However, its development is hindered by the scarcity of efficient and durable dual‐functional materials (DFMs) that combine high capture capacity with stable catalytic activity. Herein, we construct a Ni‐MgO/CaO DFM by engineering NiO‐MgO solid solution (Mg_x_Ni_1‐x_O) on a hierarchically porous CaO support incorporating highly dispersed MgO. The modulated Mg_x_Ni_1‐x_O phase fosters a strong metal‐support interaction to inhibit Ni agglomeration and carbon deposition, and simultaneously acts as a structural stabilizer to mitigate CaO sintering. The resultant material delivers a CO_2_ uptake of 11.5 mmol g^−1^, an in situ CO_2_ conversion of 90 %, and a syngas yield of 55 mmol g^−1^ at a relatively low temperature of 620°C, while exhibiting insignificant deactivation over 65 cycles, outperforming the previously developed CaO‐Ni based DFMs. Mechanistic studies reveal that this material achieves a reaction‐induced reversible reconstruction during the CaLDRM process, which not only sustains the Mg_x_Ni_1‐x_O‐mediated structural stability enhancement but also ensures the high accessibility of catalytic sites, thereby decoupling the activity‐stability trade‐off. This work provides a practical material design strategy for efficient CO_2_ capture and catalytic conversion processes.

## Graphical abstract

A highly dispersed NiO‐MgO solid solution (Mg_x_Ni_1‐x_O) is created in a hierarchically porous Ca‐based matrix to protect Ni and CaO nanoparticles against carbon deposition and sintering. Owing to the reversible reconstruction of Mg_x_Ni_1‐x_O, this superior structure is self‐regenerative in the calcium looping dry reforming of methane process, thereby achieving an unprecedented cyclic stability at competitive CO_2_ capture and in situ conversion capacity.

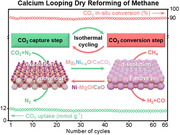

## 1 Introduction

Integrated CO_2_ capture and utilization, involving the capture of CO_2_ from hydrocarbon fuel‐dependent industrial facilities or air followed by in situ (or on‐site) valorization of the captured CO_2_ to useful products, has emerged as a promising strategy to address the rising atmospheric CO_2_ level [1, 2, 3]. Among numerous technologies, calcium looping dry reforming of methane (CaLDRM) stands out, due to its significant superiority in both environmental and economic benefits [4, 5]. This technology enables direct CO_2_ removal from large emission sources and simultaneously generates revenue by converting it with another major greenhouse gas (CH_4_) into syngas [6, 7], a mixture of H_2_ and CO that is widely used in synthesizing energy‐dense fuels and high‐value chemicals [8, 9]. The typical CaLDRM process, comprising cyclic CO_2_ capture and conversion steps, is achieved on a CaO‐Ni based dual‐functional material (DFM) in a single reactor via alternately switching feed gases. CO_2_ is first captured through the carbonation of CaO, and then the formed CaCO_3_ is decomposed in a CH_4_ atmosphere to regenerate CaO, accompanied by the catalytic reforming of CH_4_ with the released CO_2_ into syngas over Ni [10].

Owing to the opposite thermodynamic properties of CaO carbonation (exothermic) and CH_4_ dry reforming (endothermic), the CaO‐Ni based DFMs must afford both high CO_2_ uptake ability and matched catalytic activity in the same temperature range to ensure the overall CO_2_ processing capacity, consequently liberating the full potential of CaLDRM technology [11]. In this context, many efforts have been made to improve the activity of DFMs through meticulously engineering the adsorption and catalytic sites and their interfaces, achieving impressive performance, such as near‐theoretical CO_2_ uptake and ultra‐high CO_2_ conversion (≥ 90 %) [12, 13]. However, the development of CaLDRM remains hindered by the intrinsically‐low structural stability of CaO‐Ni based DFMs: (i) the difference in molar volume of CaO and CaCO_3_ induces periodic material expansion and contraction, compounded by the poor sintering resistance of CaCO_3_ (attributable to its low Tammann temperature, 533°C), easily leading to the agglomeration of CaO grains [14]; (ii) the unbalance in the reaction kinetics of CH_4_ cracking and CO_2_ activation triggers the deposition of carbon [15, 16], which rapidly grows into carbon filaments and expels Ni from the material bulk, thereby resulting in the loss of active interface [17]. These structural distortions inevitably induce the material deactivation, leading to significantly declined CO_2_ capture and conversion performance.

While incorporating a high‑Tammann‑temperature, chemically inert oxide (e.g., MgO) as a structural stabilizer into the Ca‐based matrix can effectively retard the CaO sintering during cyclic CO_2_ capture [18], this strategy inevitably comes at the expense of the material's inherent CO_2_ uptake capacity [19], since the addition of stabilizer proportionally reduces CaO content. Furthermore, the in situ exsolution of well‐dispersed Ni nanoparticles from reducible Ni‐containing oxide composites enables a strong metal‐support interaction (MSI), which significantly enhances resistance to Ni agglomeration and carbon deposition. This makes the strategy highly attractive for DRM catalyst upgrading [20, 21, 22, 23, 24, 25]. The NiO‐MgO solid solution is a particularly promising oxide composite due to its low cost and facile preparation. In this system, NiO undergoes only partial reduction, yielding exsolved Ni particles that remain semi‑embedded within the MgO matrix and thus inducing MSI. However, the means of enhancing stability by sacrificing NiO reducibility leads to a certain extent of compromise in catalytic activity [24, 26, 27]. All situations motivate us to design a novel MgO‐modified CaO‐Ni DFM capable of overcoming the aforementioned trade‐off between activity and stability.

Recently, we developed a highly dispersed MgO‐incorporated CaO hierarchically porous material (denoted as CaMg) [28], featuring all characteristics for achieving efficient and stable CO_2_ cyclic capture: (i) the porous framework (Figure S1) buffers the carbonation‐induced volumetric expansion, thereby mitigating CaCO_3_ agglomeration; (ii) the homogeneous distribution of MgO allows for its minimal use while maintaining structural stability, thus affording a high CO_2_ uptake capacity. In this work, we employed CaMg as the support to construct Ni‐MgO/CaO DFMs. A facile preparation method was developed to precisely regulate the formation of NiO‐MgO solid solution. The resultant DFMs maximally preserve the structural characteristics of the support, thereby exhibiting stable CO_2_ cyclic capture performance. The optimal material was obtained under the Ni loading of 8.7 wt. %, which not only achieves the adequate dissolution of Ni into solid solution but also generates high‐dispersion exsolved Ni nanoparticles anchored on MgO after reduction, thus ensuring both high stability and catalytic activity to DRM. Thanks to the presence of a reaction‐induced reversible reconstruction, this material can self‐regenerate its advantageous structure during the CaLDRM process, consequently maintaining exceptional CO_2_ uptake (10.0–11.5 mmol g^−1^) and in situ conversion (90 %) over 65 cycles at 620°C, superior to the previously developed CaO‐Ni based DFMs.

## 2 Results and Discussion

### 2.1 Modulation of NiO‐MgO Solid Solution

**FIGURE 1:**
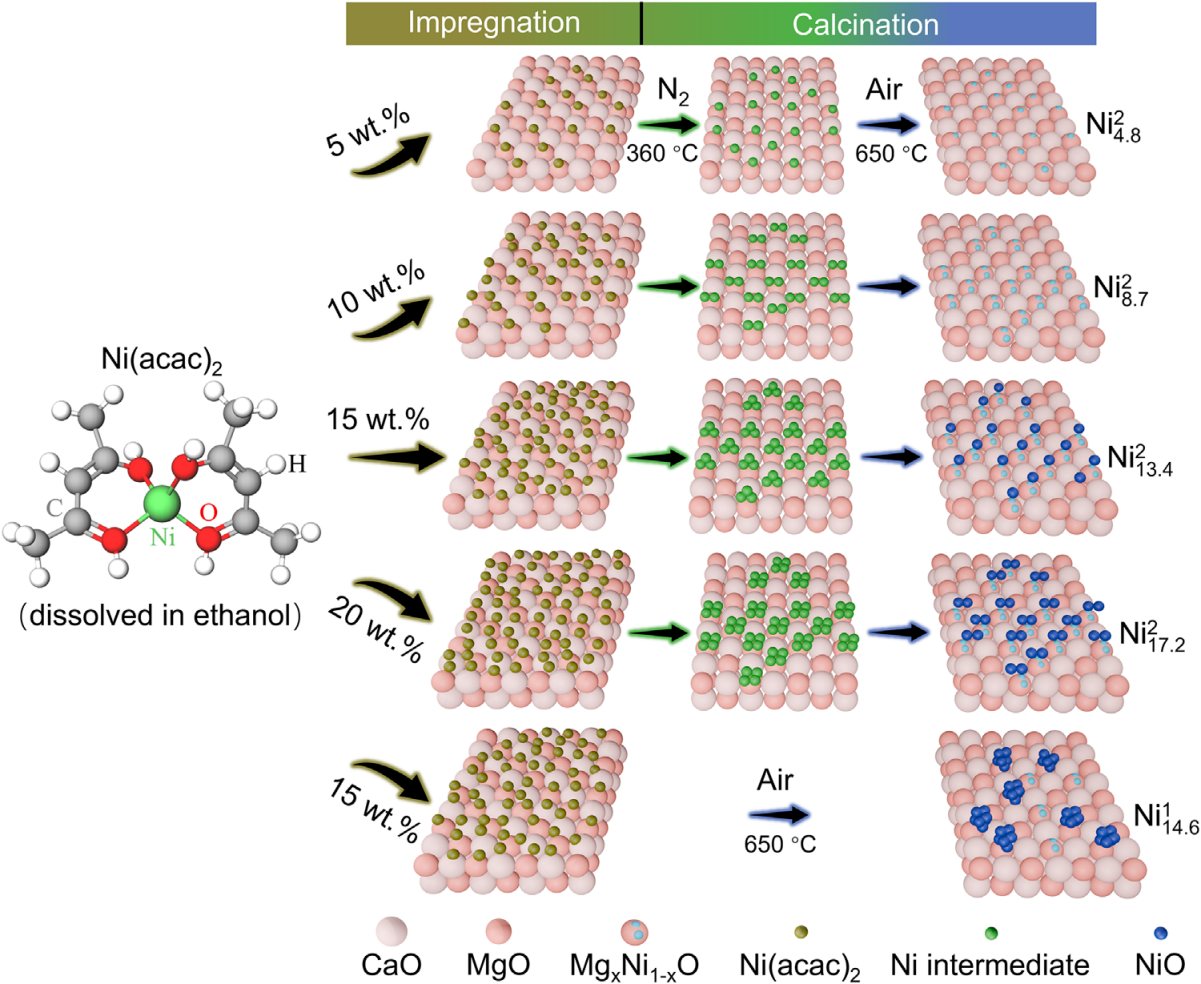
Schematics of the evolution of Ni species on CaMg support during the designed preparation process and the resultant Ni‐MgO/CaO DFMs.

The Ni‐MgO/CaO DFMs were prepared through a modified wet impregnation method using an ethanol solution of nickel acetylacetone (Ni(acac)_2_), followed by a two‐step calcination in different atmospheres (Figure 1). Impregnation with organic nickel solution could avoid structure degradation associated with the hygroscopic expansion of CaO [29, 30], so as to preserve the porous features of the CaMg support. The initial calcination temperature of 360°C is adopted because Ni(acac)_2_ could be completely decomposed at this temperature (Figures S2 and S3). The use of an N_2_ atmosphere aims to suppress grain aggregation typically induced by vigorous oxidation reactions during calcination in air [31, 32]. This facilitates the formation of uniform Ni intermediates, i.e., highly dispersed Ni particles with a metallic surface that is partially embedded within the MgO matrix (Figure S3), on the CaMg support. Owing to the similar lattice parameters of NiO (4.178 Å) and MgO (4.212 Å), as well as their shared rock‐salt crystal structure [27]. NiO and MgO readily form a solid solution. Accordingly, the second calcination is conducted at 650°C in air to fully oxidize the Ni intermediate and in situ generate a NiO‐MgO solid solution (termed as Mg_x_Ni_1‐x_O). A series of Ni‐MgO/CaO DFMs with varying Ni loading amounts was prepared by the designed method to validate its regulation on Mg_x_Ni_1‐x_O. A counterpart material was also prepared using the same method but subjected to one‐step calcination (at 650°C in air). They are denoted as ${\mathrm{Ni}}_{x}^{y}$ (Figure 1), where x represents the actual Ni content (Table S1), and y indicates the number of calcinations.

**FIGURE 2:**
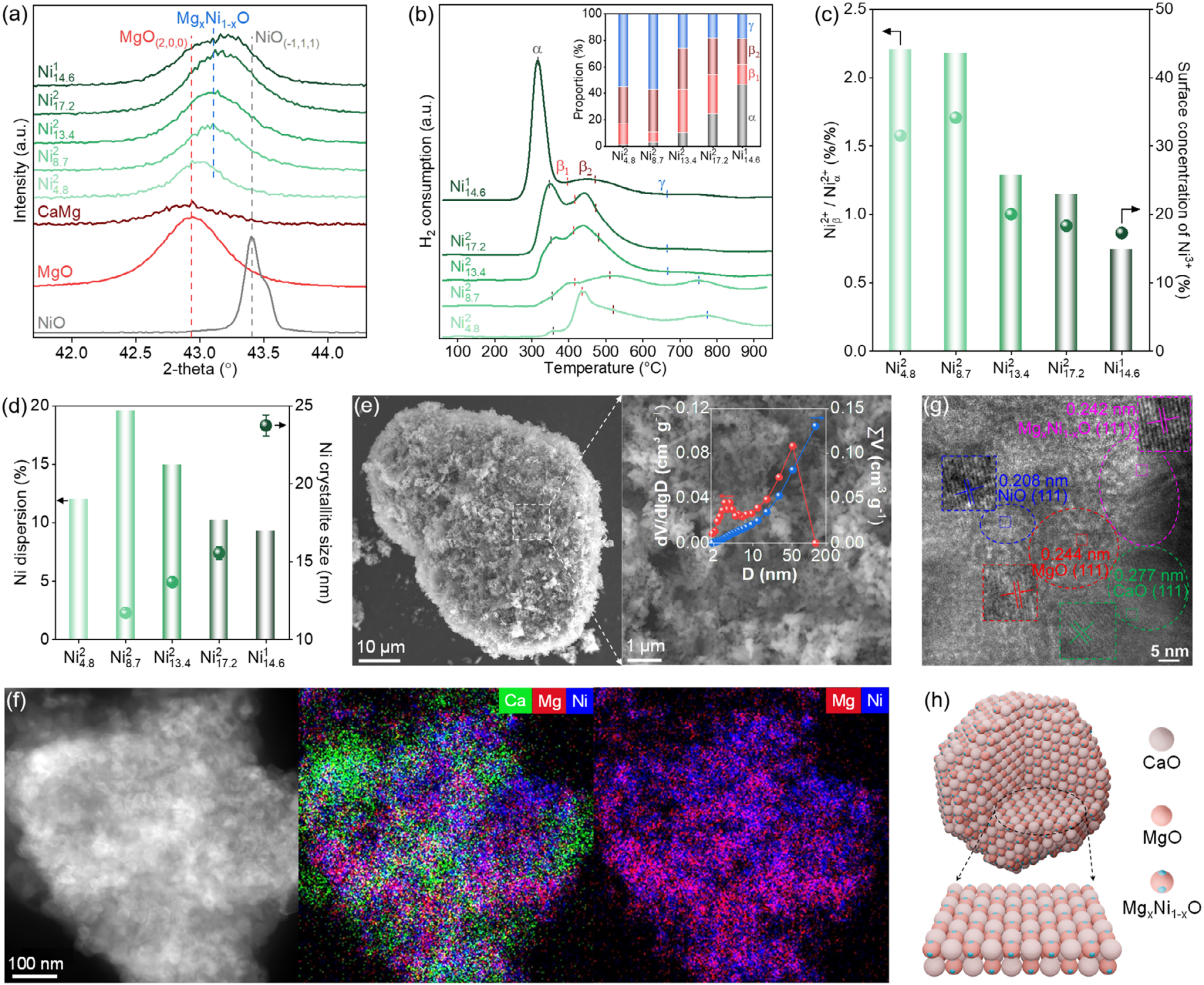
(a) Zoomed‐in XRD patterns and (b) H_2_‐TPR profiles of the as‐prepared samples; full XRD patterns are provided in Figure S4, and the position and proportion of TPR peaks are listed in Table S4. (c) The ratio of ${\mathrm{Ni}}_{\beta }^{2+}$/${\mathrm{Ni}}_{\alpha }^{2+}$ as well as the concentration of Ni^3+^ on the surface of reduced samples, determined by the fitting of XPS spectra (Figure S6). (d) The crystallite size of metallic Ni in the reduced samples and the dispersion of Ni determined by H_2_ pulse chemisorption are listed in Tables S5 and S2, respectively. (e) FESEM images and pore size distribution, (f) HAADF‐STEM image and EDS elemental maps, (g) HRTEM image, as well as (h) structural schematic of the as‐prepared ${\mathrm{Ni}}_{8.7}^{2}$.

X‐ray diffraction (XRD) patterns of the as‐prepared materials reveal that no new Ca‐related crystal phase appears in the DFMs compared to the CaMg support (Figure S4), implying the circumvention of CaO denaturation by employing organic solution‐assisted impregnation. Figure 2a displays a zoom of the XRD patterns within the region of 42°–44°. Clearly, a distinct diffraction peak positioned between the characteristic peaks of MgO and NiO is observed in the DFMs, indicating the formation of Mg_x_Ni_1‐x_O [33]. Owing to their similar lattice parameters, the diffraction peaks of MgO, Mg_x_Ni_1‐x_O, and NiO highly overlap, resulting in a merging diffraction pattern, which is largely affected by the Ni loading amount. In the material with a lower Ni content (${\mathrm{Ni}}_{4.8}^{2}$), the absence of the NiO peak alongside the presence of a shoulder peak at the MgO position indicates that Ni atoms are effectively integrated into the solid solution, leaving excess MgO. With the Ni content increases (${\mathrm{Ni}}_{4.8-17.2}^{2}$), the weakening of the MgO shoulder peak, accompanied by the enhancement of the Mg_x_Ni_1‐x_O peak, signifies the progressive development of the solid solution. However, an excess Ni loading leads to the formation of free NiO clusters, shown by the emergence of a shoulder peak at the NiO position in ${\mathrm{Ni}}_{13.4}^{2}$ and ${\mathrm{Ni}}_{17.2}^{2}$. In contrast, ${\mathrm{Ni}}_{8.7}^{2}$ exhibits an optimal composition, with Ni and Mg fully integrated into the solid solution, evidenced by the symmetrical pattern centered at the Mg_x_Ni_1‐x_O position. Despite their comparable Ni loading, ${\mathrm{Ni}}_{13.4}^{2}$ exhibits markedly smaller MgO and NiO shoulder peaks than ${\mathrm{Ni}}_{14.6}^{1}$, attesting the critical role of the two‐step calcination in facilitating the formation of solid solution. All results confirm that the designed preparation method successfully realizes the modulation of NiO‐MgO solid solution on the MgO‐incorporated CaO material, as illustrated in Figure 1.

The Mg_x_Ni_1‐x_O‐induced MSI on the Ni‐MgO/CaO DFMs was studied. H_2_ temperature‐programmed reduction (H_2_‐TPR) profiles of the as‐prepared DFMs exhibit four distinct peaks (Figure 2b). NiO particles free from interaction are easily reduced (Figure S5), yielding a peak at a low temperature range (α, 300°C–400°C). Two peaks within the relatively high temperature range (400°C–600°C) respectively originate from the reduction of the outer layer (β_1_) and the subsurface (β_2_) of the NiO interacted with the support [34]. Owing to strong interaction within the solid solution, the deeply incorporated Ni^2+^ ions in Mg_x_Ni_1‐x_O are extremely difficult to reduce, resulting in a broad peak (γ) at much higher temperatures (>600°C) [35]. Notably, the proportion of the α peak on ${\mathrm{Ni}}_{4.8-17.2}^{2}$ is much lower than that on ${\mathrm{Ni}}_{14.6}^{1}$, indicating that the two‐step calcination contributes to the formation of MSI. With the decrease of Ni loading (${\mathrm{Ni}}_{17.2}^{2}$ to ${\mathrm{Ni}}_{4.8}^{2}$). An obvious decrease in the proportion of α peak, accompanied by an increase in γ peak, reveals that the designed preparation method achieves control over MSI via regulating NiO‐MgO solid solution.

The surface composition of the DFMs was identified by X‐ray photoelectron spectra (XPS). In the Ni 2p_3/2_ spectra (Figure S6a), four overlapping peaks are respectively assigned to the metallic Ni (Ni^0^, at 852.6 eV), the Ni^2+^ in the surface (${\mathrm{Ni}}_{\alpha }^{2+}$, at 854.2 eV) and bulk (${\mathrm{Ni}}_{\beta }^{2+}$, at 855.7 eV) of NiO that interacts with the support, as well as the Ni^3+^ defects and the enrichment of surface layers by Ni^2+^ ions (Ni^3+^, at 857.1 eV) [11, 34]. The ratio of ${\mathrm{Ni}}_{\beta }^{2+}$ to ${\mathrm{Ni}}_{\alpha }^{2+}$ (${\mathrm{Ni}}_{\beta }^{2+}$/${\mathrm{Ni}}_{\alpha }^{2+}$) could serve as an indication for the strength of MSI, while the presence of Ni^3+^ defects promotes the oxygen transfer (from CO_2_ to lattice) and consequently suppresses coke formation [27, 36]. The Mg 2p spectra (Figure S6b) exhibit an obvious peak at 51.1 eV, attributed to the contribution from Mg^2+^ ions in Mg_x_Ni_1‐x_O [37]. The trend in the surface concentration of Mg_x_Ni_1‐x_O species follows ${\mathrm{Ni}}_{4.8}^{2}$ ≈ ${\mathrm{Ni}}_{8.7}^{2}$ > ${\mathrm{Ni}}_{13.4}^{2}$ > ${\mathrm{Ni}}_{17.2}^{2}$ > ${\mathrm{Ni}}_{14.6}^{1}$, and the same order is also present in the ${\mathrm{Ni}}_{\beta }^{2+}$/${\mathrm{Ni}}_{\alpha }^{2+}$ ratio and the Ni^3+^ surface concentration (Figure 2c). However, a reverse trend is found in the crystallite size of metallic Ni, i.e., ${\mathrm{Ni}}_{4.8}^{2}$ < ${\mathrm{Ni}}_{8.7}^{2}$ < ${\mathrm{Ni}}_{13.4}^{2}$ < ${\mathrm{Ni}}_{17.2}^{2}$ < ${\mathrm{Ni}}_{14.6}^{1}$ (Figure 2d). These results further confirm the strong correlation between Mg_x_Ni_1‐x_O and MSI, which directly affects the properties of catalytic sites. Specifically, the formation of Mg_x_Ni_1‐x_O solid solution enhances the MSI between Ni species and MgO, which not only promotes the formation of surface Ni^3+^ defects to improve the anti‐carbon deposition ability of DFMs, but also stabilizes the embedded Ni^2+^ ions to facilitate the exsolution of highly‐dispersed and small Ni nanoparticles during reduction (Figure 2d).

However, the reduced ${\mathrm{Ni}}_{4.8}^{2}$ shows a counterintuitive Ni dispersion, which is attributed to its limited surface active sites ― the available Ni atoms on the material surface (Table S2). Indeed, no diffraction peak corresponding to metallic Ni is observed on its XRD pattern (Figure S7), indicating that the exsolution of Ni from Mg_x_Ni_1‐x_O is limited upon reduction. In contrast, ${\mathrm{Ni}}_{8.7}^{2}$ achieves the optimal modulation of Mg_x_Ni_1‐x_O, attributable to the fact that it not only possesses strong MSI but also enables an adequate exsolution of highly dispersed Ni nanoparticles after reduction. Field‐emission scanning electron microscopy (FESEM) images combined with pore size distribution (Figure 2e) reveal that ${\mathrm{Ni}}_{8.7}^{2}$ has a similar porous morphology and porosity as CaMg (Figure S1a and Table S3), indicating the preservation of the advantageous structure for CO_2_ cyclic capture. High‐angle annular dark field‐scanning transmission electron microscopy (HAADF‐STEM) image complemented with energy dispersive spectroscopy (EDS) elemental maps shows that Ca, Mg, and Ni are homogeneously distributed in its porous framework (Figure 2f). High‐resolution transmission electron microscopy (HRTEM) image further displays that NiO is isolated from CaO by the adjacent MgO and Mg_x_Ni_1‐x_O (Figure 2g). These observations confirm that in the ${\mathrm{Ni}}_{8.7}^{2}$ DFM, Ni is anchored on MgO via the formation of Mg_x_Ni_1‐x_O solid solution, and the latter remains highly dispersed in the CaO matrix, thereby functioning as a structural stabilizer (Figure 2h).

### 2.2 CO_2_ Capture and Catalytic Performance

**FIGURE 3:**
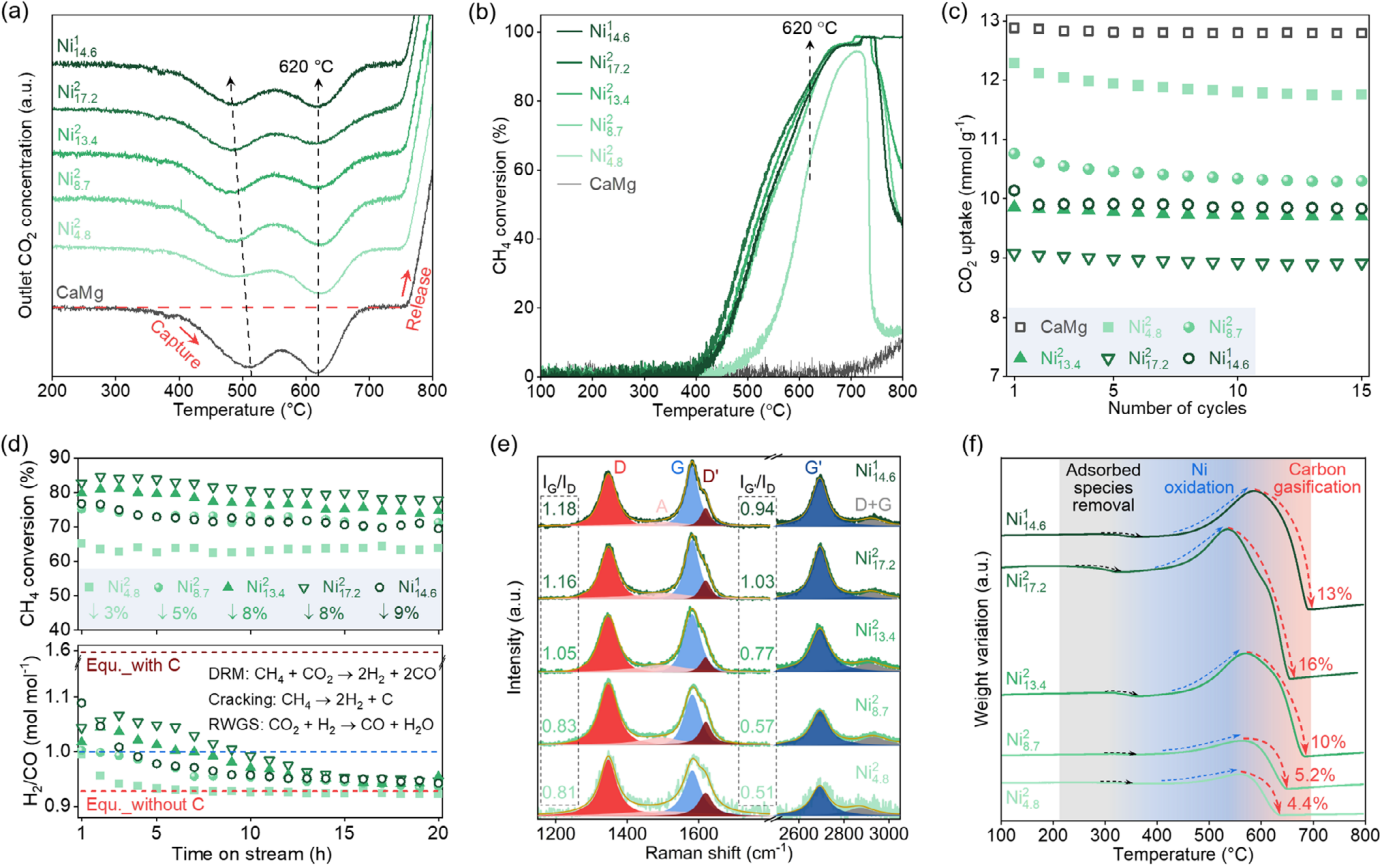
(a) CO_2_‐TPC profiles of the reduced samples obtained by treating the as‐prepared samples with 5 vol. % H_2_/Ar at 650°C for 30 min. (b) CH_4_ conversion during CH_4_‐TPSR on the carbonated samples obtained by treating the reduced samples with 5 vol. % CO_2_ at 620°C for 1 h. (c) CO_2_ uptake performance of the as‐prepared materials during 15 cycles of isothermal CO_2_ adsorption and desorption at 620°C. (d) CH_4_ conversion and the molar ratio of H_2_ to CO (H_2_/CO) during a 20 h steady‐state DRM experiment performed on the carbonated samples at 620°C; the percentages represent the deactivation degree of the DFMs after 20 h. (e) Raman spectra and (f) TGA profiles of the spent samples after 20 h DRM test; I_G_/I_D_ and I_G′_/I_D_ represent the intensity ratios of G band to D band and *G′* band to D band, respectively; TGA profiles were recorded during calcination of samples in pure air and the weight loss in carbon gasification region indicate the amount of carbon deposits.

A CO_2_ temperature‐programmed capture (CO_2_‐TPC) experiment was performed to study the dynamic CO_2_ capture‐release behavior on the reduced samples (Figure 3a), where the drop and rise of outlet CO_2_ concentration in the capture region indicate the increase and decrease of CO_2_ uptake rate, respectively. All materials exhibit two CO_2_ uptake peaks, ascribed to the carbonation of surface (350°C–550°C) and internal (550°C–700°C) CaO. The first peak slightly shifts toward lower temperature with loading of Ni, indicating the promotion of surface CaO carbonation, which is highly relevant to the enhanced surface alkalinity in the presence of metallic Ni (Figure S8). The second one is largely affected by the diffusion of CO_2_ in the network of pores and the newly formed CaCO_3_ layer, which depends on the mesoporosity and grain size [28]. Therefore, the similar temperature required for internal CaO carbonation is attributed to their comparable porosity (Table S3) and CaO crystallite size (Table S6). Notably, at 620°C, all materials reach the maximum CO_2_ uptake rate before CO_2_ release. CH_4_ temperature‐programmed surface reaction (CH_4_‐TPSR) was conducted to investigate the CH_4_ activation on the carbonated samples (Figure S9). On the DFMs, the drop of CH_4_ accompanied by an instantaneous rise of H_2_ at around 400°C signifies the emergence of CH_4_ cracking.

Subsequently, the rise of CO and CO_2_ is observed at around 550°C, indicating the initiation of CH_4_ dry reforming. On the CaMg support, all the above phenomena are not observed, except a rise of CO_2_ (at 600°C) originating from the decomposition of CaCO_3_. Thus, the temperatures suitable for the CO_2_ conversion step of CaLDRM implemented on the as‐prepared DFMs should be beyond 600°C, at which CaCO_3_ decomposition and DRM reaction can be effectively coupled. Here, we adopt 620°C as the operating temperature of the CaLDRM process, which allows for achieving efficient CO_2_ capture (Figure 3a) and significant CH_4_ conversion (Figure 3b) simultaneously.

Next, the CO_2_ capture performance of the DFMs at 620°C was examined. All DFMs maintain over 95 % of the initial CO_2_ uptake after 15 cycles of isothermal CO_2_ adsorption and desorption (Figure 3c; Figure S10), demonstrating a stable CO_2_ cyclic capture. Additionally, they exhibit almost unchanged crystallite size of CaO after 15 cycles (Figures S11 and S12), confirming the absence of CaO sintering. The exceptional stability of DFMs in CO_2_ cyclic capture benefits from their advantageous structure (i.e., small CaO grain, macropores, and highly‐dispersed MgO incorporation) retrieved from the CaMg support (Tables S6 and S3 and Figure S13; Figure 2f) [28]. Given that all DFMs exhibit excellent cyclic stability, their CO_2_ capture performance can be directly assessed by the CO_2_ uptake, which follows the order of ${\mathrm{Ni}}_{4.8}^{2}$ > ${\mathrm{Ni}}_{8.7}^{2}$ > ${\mathrm{Ni}}_{13.4}^{2}$ ≈ ${\mathrm{Ni}}_{14.6}^{1}$ > ${\mathrm{Ni}}_{17.2}^{2}$ (Figure 3c), consistent with the trend in CaO content (Table S1). Indeed, the introduction of Ni reduces the proportion of CaO in the DFM, thereby lowering its inherent CO_2_ capture capacity.

We further compared the catalytic DRM performance of the DFMs at 620°C. The occurrence of dynamic CaO carbonation and CaCO_3_ decomposition leads to an oscillating CO_2_ conversion throughout the 20 h test (Figure S14), which does not reflect the material's real catalytic activity. The latter, however, can be determined by the CH_4_ conversion, since it is correlated to the amount of catalytic sites (i.e., surface active sites) on the materials (Table S2). As seen in Figure 3d, the catalytic activity of the DFMs follows the order as ${\mathrm{Ni}}_{4.8}^{2}$ << ${\mathrm{Ni}}_{8.7}^{2}$ ≈ ${\mathrm{Ni}}_{14.6}^{1}$ < ${\mathrm{Ni}}_{13.4}^{2}$ < ${\mathrm{Ni}}_{17.2}^{2}$. The H_2_/CO molar ratios lie between the equilibrium values calculated with (Equ._with C) and without (Equ._without C) carbon formation, implying the deposition of carbon originating from CH_4_ cracking. This is further supported by the carbon balance (Figure S15), which remains below 100 %. On ${\mathrm{Ni}}_{13.4}^{2}$, ${\mathrm{Ni}}_{17.2}^{2}$ and ${\mathrm{Ni}}_{14.6}^{1}$, H_2_/CO exceeds 1 (the stoichiometric value of the DRM reaction) during the initial hours (Figure 3d), indicating that CH_4_ cracking dominates over the reverse water‐gas shift (RWGS) reaction in this period. Subsequently, H_2_/CO drops below 1 alongside an increase in carbon balance, reflecting the weakening of CH_4_ cracking and the growing contribution of RWGS. In contrast, for ${\mathrm{Ni}}_{4.8}^{2}$ and ${\mathrm{Ni}}_{8.7}^{2}$, H_2_/CO never surpasses 1, suggesting that RWGS consistently prevails as the dominant side reaction throughout the steady‐state DRM test. Over time, H_2_/CO gradually declines toward the Equ._without C, while the carbon balance approaches 100 %, pointing the cessation of CH_4_ cracking. These observations collectively indicate that on DFMs with high Ni loading, the elevated CH_4_ conversion likely stems from severe CH_4_ cracking, which in turn implies more pronounced carbon deposition.

Raman spectra of the deposited carbon on the spent DFMs exhibit six distinct bands (Figure 3e), arising from the disordered and defective structures (D at 1348 cm^−1^, *D′* at 1620 cm^−1^), amorphous carbon (A at 1495 cm^−1^), graphitic nature (G at 1582 cm^−1^), two‐photon elastic scattering process on the layered or nanotubular structures (*G′* at 2693 cm^−1^), and combination of the D and G resonances (D+G at 2930 cm^−1^), respectively [46, 47]. The presence of a significant single peak at the *G′* band indicates the formation of carbon nanotubes (i.e., carbon filaments). Generally, a higher I_G_/I_D_ ratio indicates a higher graphitization degree of the deposited carbon, while the I_G′_/I_D_ ratio is highly related to the purity of carbon filaments, as the two‐photon elastic scattering process would not occur in disordered structures [48]. Therefore, higher I_G_/I_D_ and I_G′_/I_D_ ratios signify the formation of more high‐quality carbon filaments, which have lower reactivity to oxidant (e.g., CO_2_) [49], culminating in more severe carbon deposition during DRM (Figure 3f). In addition, all DFMs show increased Ni crystallite sizes after 20 h steady‐state DRM (Figures S16 and S17), indicating the occurrence of Ni sintering. Deposited carbon can not only block catalytic sites but also reduce active interfaces by separating Ni particles from the CaMg support, ultimately causing catalytic performance degradation [21, 30]. Meanwhile, the sintering of Ni nanoparticles, which also leads to the loss of catalytic sites, represents another factor contributing to performance degradation [21]. Consequently, the deactivation degree of the DFMs (Figure 3d), which follows the order of ${\mathrm{Ni}}_{4.8}^{2}$ < ${\mathrm{Ni}}_{8.7}^{2}$ < ${\mathrm{Ni}}_{13.4}^{2}$ ≈ ${\mathrm{Ni}}_{17.2}^{2}$ < ${\mathrm{Ni}}_{14.6}^{1}$, correlates strongly with both the amount of carbon deposition (Figure 3f) and the extent of Ni sintering (Figure S17).

Based on a comprehensive evaluation of CO_2_ cyclic capture and steady‐state DRM performance, ${\mathrm{Ni}}_{8.7}^{2}$ is identified as the optimal DFM. It maintains a relatively high CaO content, ensuring considerable CO_2_ capture capacity, while achieving rational modulation of the Mg_x_Ni_1‐x_O phase. Specifically, sufficient Ni dissolution into the solid solution establishes a strong metal‐support interaction (MSI) that inhibits Ni nanoparticle migration and promotes the formation of Ni^3+^ defects favorable for oxygen transfer, thereby suppressing Ni sintering and carbon deposition. Meanwhile, effective Ni exsolution upon reduction provides ample surface active sites, enabling comparable catalytic activity. Although ${\mathrm{Ni}}_{13.4}^{2}$ and ${\mathrm{Ni}}_{17.2}^{2}$ exhibit higher catalytic activity than ${\mathrm{Ni}}_{8.7}^{2}$, their low CO_2_ uptake (due to reduced CaO content) and poor resistance to carbon deposition and Ni sintering (arising from insufficient Ni dissolution in the solid solution and the consequent weak MSI) preclude them from enabling an efficient and stable CaLDRM process. In contrast, ${\mathrm{Ni}}_{4.8}^{2}$ shows slightly better anti‐carbon deposition ability and higher CO_2_ uptake than ${\mathrm{Ni}}_{8.7}^{2}$, but its substantially lower catalytic activity, resulting from limited Ni exsolution upon reduction, disqualifies it as a suitable DFM.

### 2.3 Long‐Term CaLDRM Performance

**FIGURE 4:**
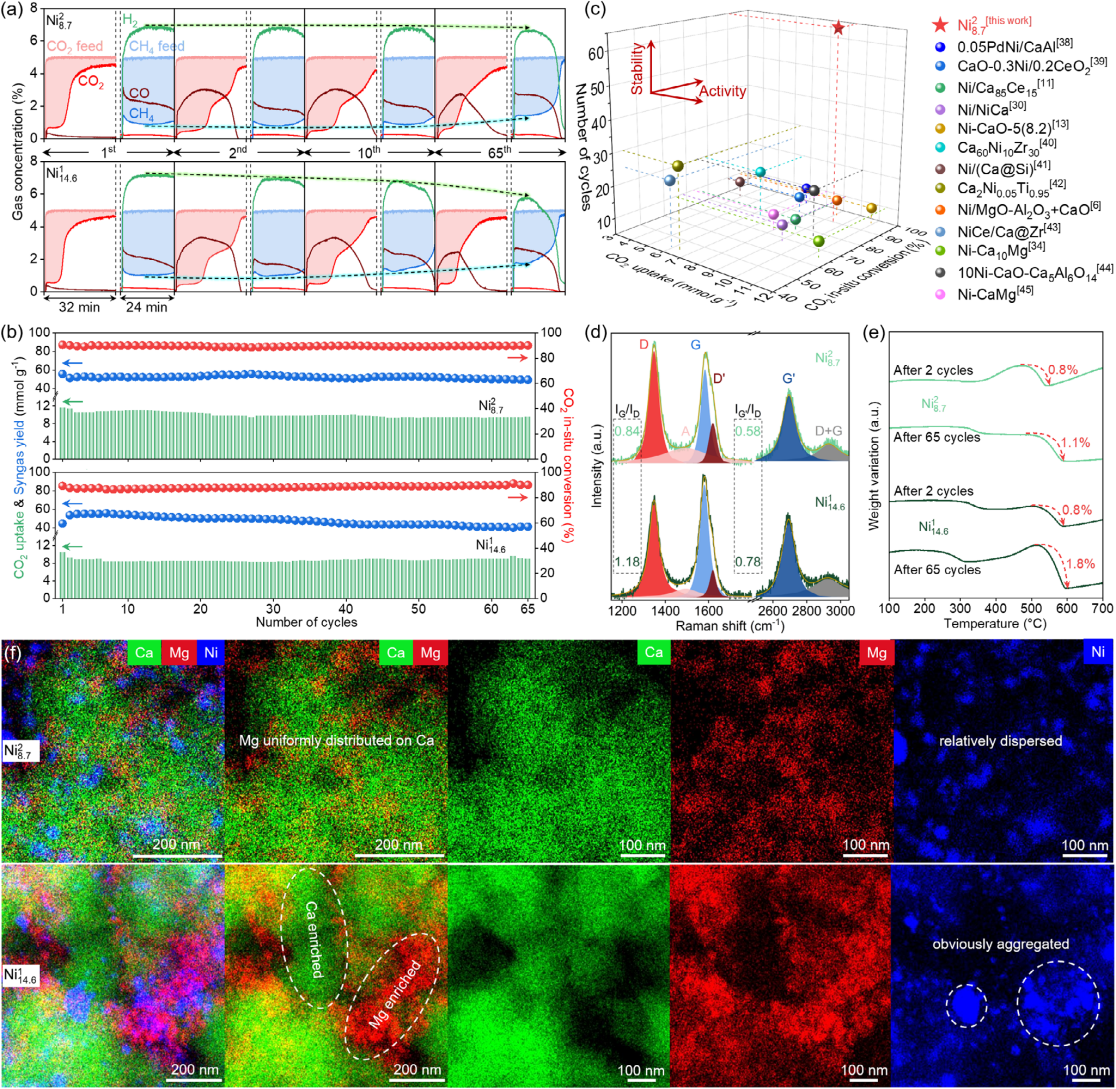
(a) The concentrations of inlet (feed) and outlet (product) gases (four representative cycles are displayed) as well as (b) CO_2_ uptake, CO_2_ in situ conversion, and syngas yield during 65 cycles of CaLDRM implemented on the reduced ${\mathrm{Ni}}_{8.7}^{2}$ and ${\mathrm{Ni}}_{14.6}^{1}$ DFMs at 620°C; one cycle comprises 32 min CO_2_ capture step (in 5 vol. % CO_2_/Ar), 24 min CO_2_ conversion step (in 5 vol. % CH_4_/Ar), and 2 min Ar purging in between. (c) Comparison of the activity and stability of CaO‐Ni based DFMs in the literature and this work [6, 11, 13, 30, 34, 38, 39, 40, 41, 42, 43, 44, 45]; the CO_2_ uptake and in situ conversion presented are the values in the last cycle (Table S7). (d) Raman spectra, (e) TGA profiles, and (f) STEM‐EDS elemental maps of the samples after 65 cycles of CaLDRM (end with the CO_2_ conversion step).

The performance of ${\mathrm{Ni}}_{8.7}^{2}$ in the long‐term CaLDRM process was assessed by performing 65 repeated cycles at 620°C. To highlight the enhanced stability of ${\mathrm{Ni}}_{8.7}^{2}$, ${\mathrm{Ni}}_{14.6}^{1}$ was employed for comparison, because it matches the activity of ${\mathrm{Ni}}_{8.7}^{2}$ in CO_2_ capture and catalytic DRM but exhibits poorer resistance to carbon deposition and Ni sintering. Figure 4a displays the compositions of inlet and outlet gases in different cycles, where the shadow regions indicate the consumption of CO_2_ or CH_4_. Overall, both CO_2_ capture and conversion initiate immediately upon switching to the respective feed gases. During the CO_2_ conversion step, CH_4_ is in situ reformed with the released CO_2_ to yield H_2_ and CO, whereby the significant excess of H_2_ over CO indicates the simultaneous occurrence of CH_4_ cracking. Subsequently, the generated carbon is gasified through the reverse Boudouard reaction (C + CO_2_ → 2CO) during the next CO_2_ capture step, as evidenced by the formation of CO.

Figure 4b provides the quantitative results of the material performance. Both ${\mathrm{Ni}}_{8.7}^{2}$ and ${\mathrm{Ni}}_{14.6}^{1}$ enable stable CO_2_ capture in cyclic CaLDRM process, with a capacity retention exceeding 85 % after 65 cycles, while the former (11.5 mmol g^−1^) affords a higher initial CO_2_ uptake than the latter (10.5 mmol g^−1^). Moreover, they sustain a considerable in situ CO_2_ conversion (90 %) throughout 65 cycles without any decline. The strong endothermicity of CaCO_3_ decomposition induces a slow and steady CO_2_ release at 620°C, which allows for sufficient reaction with CH_4_ [6], leading to high CO_2_ conversion and consequently high syngas yield (up to 55 mmol g^−1^). The changes of outlet H_2_ and CH_4_ concentrations with cycles clearly indicate a difference in their catalytic stability (Figure 4a). After 65 cycles (Figure 4b), ${\mathrm{Ni}}_{8.7}^{2}$ holds 90 % of its initial syngas yield, in contrast, ${\mathrm{Ni}}_{14.6}^{1}$ presents a substantial drop by 30 %, indicating higher catalytic stability of the former, consistent with the results revealed by CH_4_ conversion (Figure S18). To the best of our knowledge, ${\mathrm{Ni}}_{8.7}^{2}$ outperforms all reported CaO‐Ni based DFMs in the CaLDRM process (Figure 4c), which is ascribed to its exceptional activity and cyclic stability achieved at a relatively low temperature (Figure S19).

The post‐reaction samples were subjected to comprehensive characterization to probe their structural changes. Compared to the deposited carbon after 20 h steady‐state DRM (Figure 3e), that after 65‐cycle CaLDRM (Figure 4d) exhibits a similar graphitization degree (as indicated by I_G_/I_D_ ratio). Carbon filaments (identified by the *G′* peak) still form with comparable purity (reflected by I_G′_/I_D_ ratio) on ${\mathrm{Ni}}_{8.7}^{2}$, but with lower purity on ${\mathrm{Ni}}_{14.6}^{1}$. In addition, the amount of deposited carbon after 65‐cycle CaLDRM (Figure 4e) is considerably lower than that after 20 h steady‐state DRM (Figure 3f), owing to the periodic gasification of carbon in the CO_2_ capture step (Figure 4a). The detected carbon is thus primarily generated in the CO_2_ conversion step of the last cycle. Nevertheless, the graphitization degree and purity of the carbon filaments formed on ${\mathrm{Ni}}_{8.7}^{2}$ remain substantially lower than those on ${\mathrm{Ni}}_{14.6}^{1}$ (Figure 4d; Figure S20), suggesting that they are more readily eliminated by CO_2_, leading to less accumulation over CaLDRM cycles. Consequently, ${\mathrm{Ni}}_{8.7}^{2}$ exhibits a smaller increase in carbon deposition from the second to the 65th cycle compared to ${\mathrm{Ni}}_{14.6}^{1}$ (Figure 4e). STEM‐EDS elemental mapping indicates that ${\mathrm{Ni}}_{8.7}^{2}$ preserves the homogeneous distribution of Mg and Ni on the Ca matrix throughout the 65‐cycle CaLDRM. In contrast, ${\mathrm{Ni}}_{14.6}^{1}$ exhibits pronounced segregation of Mg and Ca alongside severe Ni aggregation after the same cycles (Figure S21; Figure 4f). SEM images (Figure S22) and crystallite sizes (Table S5) further confirm that ${\mathrm{Ni}}_{8.7}^{2}$ retains significantly smaller Ni particles than ${\mathrm{Ni}}_{14.6}^{1}$ after 65 cycles. All results demonstrate that the excellent stability of ${\mathrm{Ni}}_{8.7}^{2}$ in long‐term CaLDRM process is attributed to its superior resistance to carbon deposition and particle sintering. This is derived from its durable characteristic structure comprising a highly dispersed stabilizer‐incorporated, hierarchically porous framework and a Mg_x_Ni_1‐x_O solid solution‐mediated strong metal‐support interaction (Figure 2).

### 2.4 Structural Durability Mechanism

**FIGURE 5:**
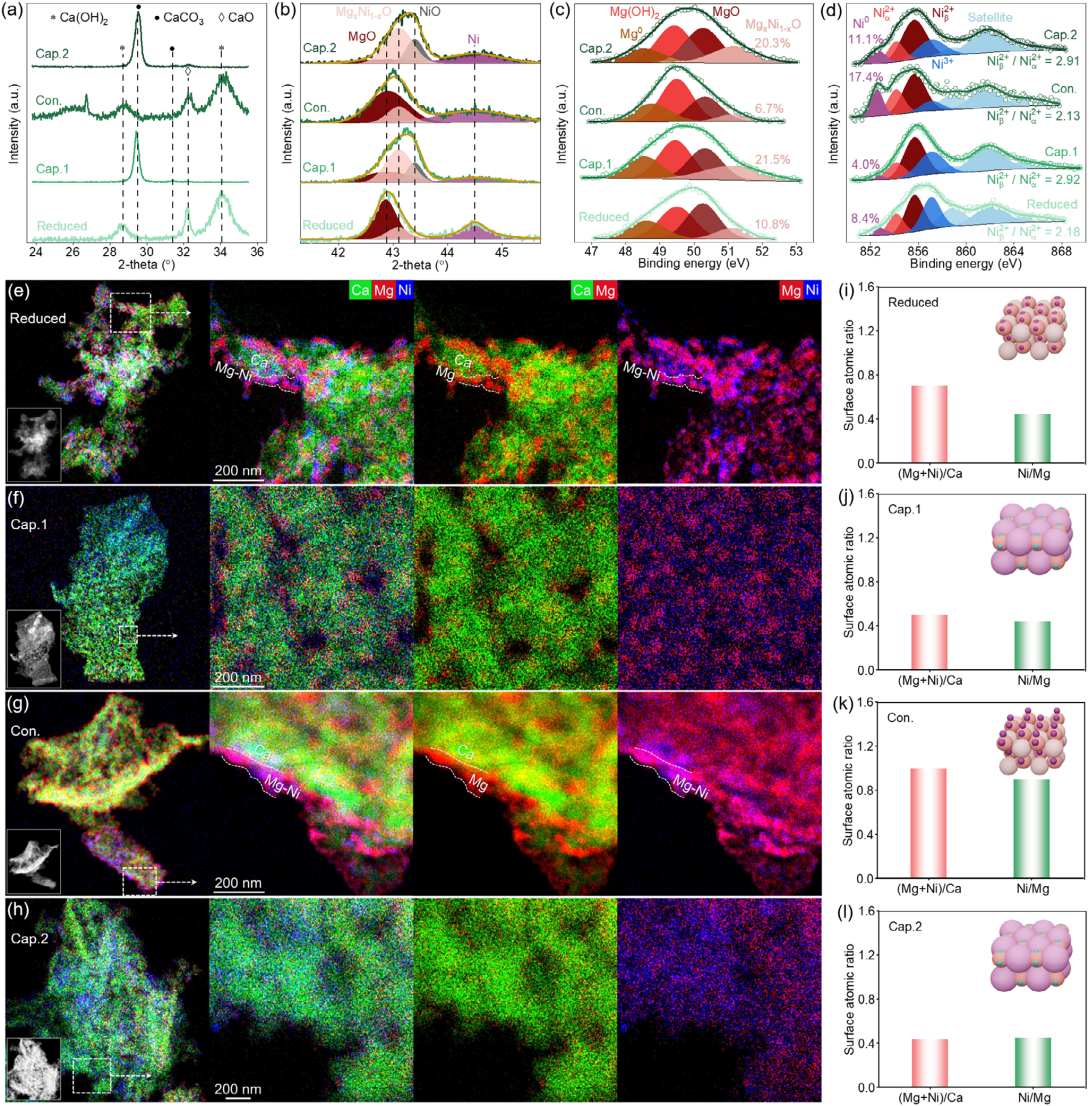
(a,b) Zoomed‐in XRD patterns, (c,d) XPS spectra and the corresponding fitting curves, (e–h) STEM‐EDS elemental maps, as well as (i–l) surface atomic ratios for ${\mathrm{Ni}}_{8.7}^{2}$ obtained from different steps in CaLDRM. Full XRD patterns are provided in Figure S23; the percentages shown in XPS spectra are the surface concentrations of Mg_x_Ni_1‐x_O and Ni^0^ species calculated based on the fitting results; (Mg+Ni)/Ca and Ni/Mg respectively represent the surface atomic ratios of (Mg+Ni) to Ca and Ni to Mg, determined by SEM‐EDS analysis (Figures S24–S27); Reduced, Cap.1, Con. and Cap.2 refer to the reduced sample, and the samples after the first CO_2_ capture, the subsequent CO_2_ conversion and the second CO_2_ capture steps, respectively.

The origin of the structural durability of ${\mathrm{Ni}}_{8.7}^{2}$ was explored through characterizing the samples derived from each step of CaLDRM. As the initial state, CaO, Ca(OH)_2_, MgO, Mg_x_Ni_1‐x_O, and metallic Ni exist in the reduced sample (Figure 5a,b). After the first CO_2_ capture step, all Ca‐related phases fully transform to CaCO_3_. Additionally, the intensities of metallic Ni and MgO diminish, accompanied by the emergence of NiO and the enhanced Mg_x_Ni_1‐x_O, signifying the oxidation of Ni and the proliferation of NiO‐MgO solid solution. This is further supported by the lower surface concentration of Ni^0^ and higher surface concentration of Mg_x_Ni_1‐x_O on the cap.1 sample, compared to the reduced sample (Figure 5c,d). Following the subsequent CO_2_ conversion step (in CH_4_), CaCO_3_ disappears, and the Ca‐related crystal phases retrieve the initial state. The increase in the intensities of metallic Ni and MgO, concurrent with the disappearance of NiO and decrease of Mg_x_Ni_1‐x_O, indicates the reduction of NiO on both the surface and the solid solution, as equally evidenced by the respective increase and decrease of Ni^0^ and Mg_x_Ni_1‐x_O surface concentrations in the con. sample. After the second CO_2_ capture step, the crystal phase transformation observed replicates that of the first capture step.

Furthermore, Mg and Ni experience a dynamic and reversible relocation within the Ca‐based matrix (Figure 5e‐h). They initially distribute on the exterior of the matrix, while transferring to the interior after the first CO_2_ capture step and returning to the exterior after the subsequent conversion step. Following the second CO_2_ capture step, Mg and Ni redistribute into the interior of the Ca matrix, consistent with that observed in the first capture step. This relocation is equally evidenced by the corresponding oscillations of the surface (Mg+Ni)/Ca atomic ratio (Figure 5i‐l), i.e., 0.70 → 0.50 → 0.99 → 0.44. Notably, Mg and Ni remain in close integration across the entire CaLDRM process, as seen in their overlapping map (Figure 5e‐h), attesting to the effective anchoring of Ni species by the solid solution. The surface Ni/Mg atomic ratio exhibits a periodic oscillation throughout successive steps (cap.1: 0.44 → con.: 0.90 → cap.2: 0.45), indicating that Ni species diffuse outward from the parent solid solution during CO_2_ conversion and reintegrate inward following CO_2_ capture.

**FIGURE 6:**
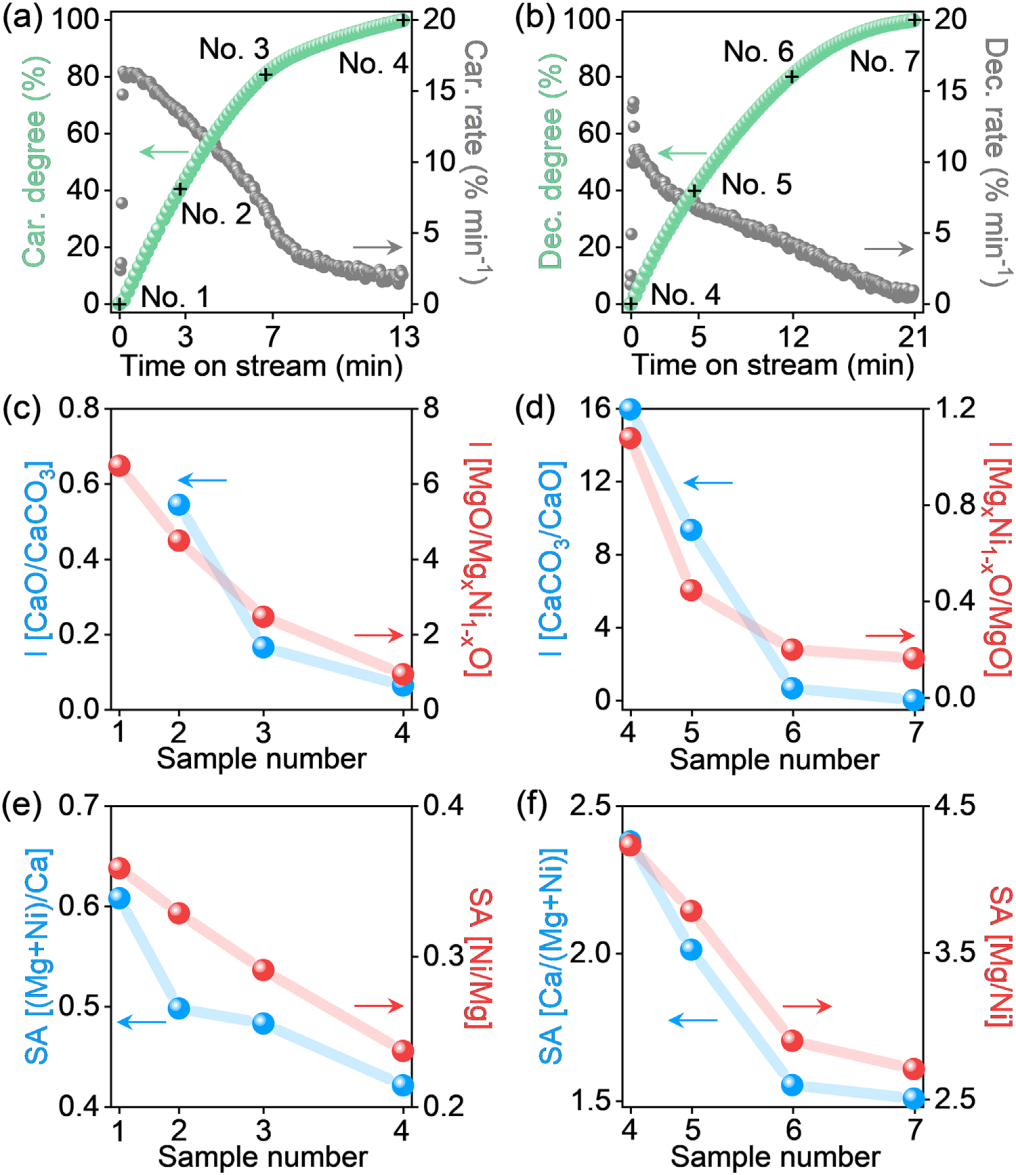
(a,b) Evolution of the carbonation (car.) and decarbonation (dec.) degrees and their corresponding rates for ${\mathrm{Ni}}_{8.7}^{2}$ during the CO_2_ capture and subsequent conversion steps in one CaLDRM cycle, calculated from inlet and outlet gas concentrations (Figure S29). No. 1–7 denote the samples collected at different reaction stages, each corresponding to a specific carbonation or decarbonation degree (i.e., 0 %, 40 %, 80 % and 100 %). Sample No. 4 was taken at the end of the CO_2_ capture step, which also served as the starting point for the subsequent CO_2_ conversion step. (c,d) Relative intensities (I) between CaO and CaCO_3_ as well as MgO and Mg_x_Ni_1‐x_O phases in the different‐stage samples, derived from the fitted area of the characteristic XRD peaks (Figure S30). Note that I[CaO/CaCO_3_] for sample No. 1 is not shown because the CaCO_3_ phase is absent. (e,f) Surface atomic ratios (SA) of (Mg+Ni) and Ca, as well as Ni and Mg for the different‐stage samples, were determined by SEM‐EDS analysis (Figure S31).

The above observations demonstrate that a reaction‐induced reversible reconstruction, involving phase transformation (Figure 5a‐d) and phase migration (Figure 5e‐l), occurs on ${\mathrm{Ni}}_{8.7}^{2}$ in the CaLDRM process. To further confirm this dynamic behavior, we compared the rates of chemical reaction and structural reconstruction by tracking the phase evolution of ${\mathrm{Ni}}_{8.7}^{2}$ at progressive carbonation and decarbonation levels (Figure 6). As shown in Figure 6a,b, ${\mathrm{Ni}}_{8.7}^{2}$ achieves complete carbonation and decarbonation during the CO_2_ capture and subsequent conversion steps. The corresponding rates rise sharply at the onset and then gradually decline, following the typical calcium‐looping kinetics — a fast reaction‐controlled regime followed by a slow diffusion‐controlled regime [28].

During CO_2_ capture step (increasing carbonation degree, Figure 6a), the relative intensities of CaO to CaCO_3_ (I[CaO/CaCO_3_]) and MgO to Mg_x_Ni_1‐x_O (I[MgO/Mg_x_Ni_1‐x_O]) decrease (Figure 6c), while the surface atomic ratios of (Mg+Ni) to Ca (SA[(Mg+Ni)/Ca]) and Ni to Mg (SA[Ni/Mg]) also drop (Figure 6e). These trends evidence the conversion of CaO to CaCO_3_ and the concurrent formation of the Mg_x_Ni_1‐x_O solid solution, which enables inward migration of Mg and Ni into the Ca‐based matrix and dissolution of Ni into the solid solution. During the subsequent CO_2_ conversion step (increasing decarbonation degree, Figure 6b), the declines in I[CaCO_3_/CaO] and I[Mg_x_Ni_1‐x_O/MgO] (Figure 6d), together with the decreases in SA[Ca/(Mg+Ni)] and SA[Mg/Ni] (Figure 6f), signal CaCO_3_ decomposition and disintegration of the solid solution. This is accompanied by outward migration of Mg and Ni from the Ca matrix and exsolution of Ni from the solid solution.

The decreasing rates of the relative intensities of the CaO‐CaCO_3_ pair and the surface atomic ratios of (Mg+Ni)‐Ca pair (all blue curves in Figure 6c‐f) follow a rapid‐to‐slow trend that well matches the evolution of the carbonation and decarbonation rates (Figure 6a,b). This consistency indicates that the CaO ↔ CaCO_3_ phase transformation, and the resulting migration of Mg and Ni within the Ca matrix, are predominantly governed by the carbonation and decarbonation reactions. Notably, during the CO_2_ capture step, the decreasing rates of I[MgO/Mg_x_Ni_1‐x_O] and SA[Ni/Mg] remain nearly constant. This implies that the formation of the solid solution (proceeds at a steady rate) is controlled by the step duration and the available Ni supply, rather than by the carbonation rate. In contrast, during the CO_2_ conversion step, the decreasing rates of I[Mg_x_Ni_1‐x_O/MgO] and SA[Mg/Ni] correlate strongly with the decarbonation rate. This observation suggests that the reduction‐induced exsolution of Ni is regulated by the decarbonation reaction, as it dictates the production of reducing gases (H_2_ and CO).

**FIGURE 7:**
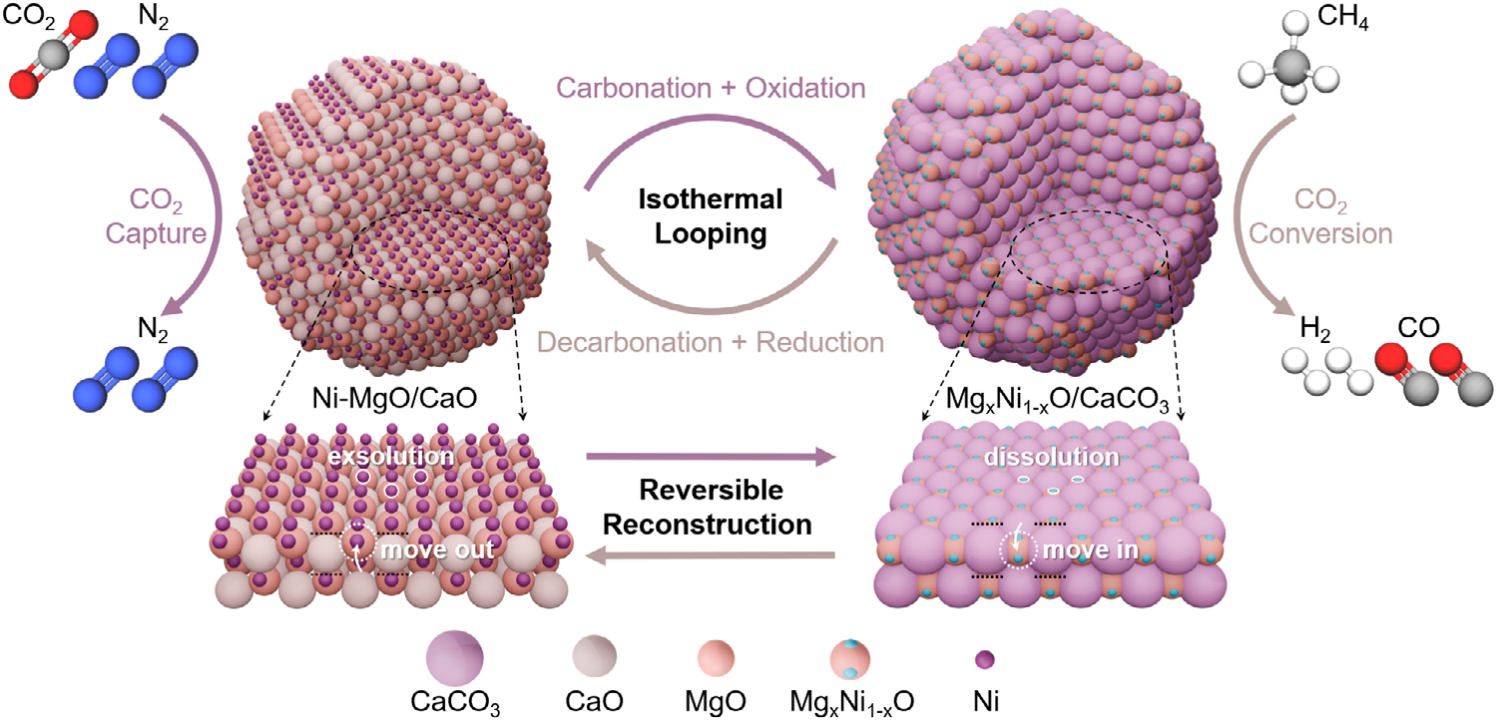
Schematic representation for the reversible reconstruction of the developed Ni‐MgO/CaO DFM during the CaLDRM process.

Through exploring the reaction‐induced reversible reconstruction behavior, a structural durability mechanism is proposed for the ${\mathrm{Ni}}_{8.7}^{2}$ DFM, as illustrated in Figure 7. During the CO_2_ capture step, carbonation of CaO to CaCO_3_ (with volumetric expansion) occurs alongside oxidation of metallic Ni to NiO, which subsequently forms Mg_x_Ni_1‐x_O solid solution with MgO. The dissolution of Ni into this solid solution establishes a strong metal‐support interaction, which protects Ni from sintering and carbon deposition. Concurrently, the Mg_x_Ni_1‐x_O phase migrates into the expanding framework, serving as a structural stabilizer that inhibits CaCO_3_ agglomeration. In the following CO_2_ conversion step, CaCO_3_ decomposes to CaO in CH_4_ (decarbonation), producing H_2_ and CO. The attendant volumetric contraction drives the outward migration of Mg_x_Ni_1‐x_O from the CaO matrix, accompanied by Ni exsolution from the solid solution under the reducing atmosphere, thereby regaining highly accessible catalytic sites. Collectively, this reversible reconstruction endows ${\mathrm{Ni}}_{8.7}^{2}$ with a self‐regenerative architecture during CaLDRM, overcoming the activity‐stability trade‐off and enabling a prolonged operational lifetime.

The intrinsic thermal coupling between the exothermic CaO carbonation and the endothermic CaCO_3_ decomposition and DRM creates a natural opportunity for heat integration. For instance, in circulating fluidized‐bed or dual fixed‐bed reactor configurations, the heat released during the CO_2_ capture step can be directly utilized to drive the heat‐demanding reactions in the CO_2_ conversion step, thereby reducing the external energy input required for the CaLDRM process [50]. In this context, ${\mathrm{Ni}}_{8.7}^{2}$ is a particularly well‐suited DFM. Its structural robustness, arising from self‐regeneration, prevents the sintering of CaO and Ni nanoparticles and minimizes the formation of carbon deposits, which would otherwise lower the carbonation and decarbonation rates and reduce the sorbent‐catalyst interfaces, thereby disrupting the thermal balance between the exothermic and endothermic steps and compromising heat transfer efficiency. Therefore, this material, with a self‐regenerative architecture enabled by reaction‐induced reversible reconstruction, not only offers a promising concept for designing highly active and stable CaO‐Ni based DFMs but also provides a practical materials model for realizing energy‐efficient CaLDRM processes with effective heat integration.

## 3 Conclusion

In summary, we designed a tailored preparation strategy, involving organic solution‐assisted impregnation followed by two‐step atmosphere‐controlled calcination, to load Ni onto a hierarchically porous CaO support incorporating highly dispersed MgO, constructing high‐performance Ni‐MgO/CaO DFMs for the CaLDRM ― a process that realizes CO_2_ capture and in situ conversion. This preparation protocol preserves the intrinsic structural characteristics of the support and permits precisely regulating the formation of Mg_x_Ni_1‐x_O solid solution via controlled Ni loading amount and first‐step N_2_ calcination temperature. The formed Mg_x_Ni_1‐x_O phase serves as a structural stabilizer within the porous framework to inhibit CaO sintering, thereby ensuring cyclically stable CO_2_ capture. Meanwhile, it establishes a strong interaction between Ni species and MgO, which not only provides stable anchoring sites to prevent the exsolved Ni from agglomeration but also promotes the formation of surface Ni^3+^ defects to mitigate carbon deposition, ultimately enhancing the catalytic DRM performance. The optimized Ni‐MgO/CaO DFM (with the Ni loading of 8.7 wt. %) exhibits considerable CO_2_ capture and conversion capacity as well as unprecedented stability in long‐term CaLDRM cycles. Comprehensive characterization reveals a reaction‐induced reversible reconstruction mechanism, wherein the Mg_x_Ni_1‐x_O phase undergoes cyclic inward and outward migration within the Ca‐based framework, accompanied by reversible dissolution and exsolution of Ni. This dynamic behavior sustains the role of Mg_x_Ni_1‐x_O in suppressing sintering and carbon deposition while cyclically regenerating accessible catalytic sites, which are the origins of the material's exceptional stability. Utilizing the reversible reconstruction of solid solution to achieve the periodic self‐regeneration of active sites could provide a promising avenue to overcome the longstanding activity‐stability trade‐off in the CO_2_ capture and in situ conversion processes.

## Author Contributions

Jiawei Hu and Wei Wei conceived the research. Hao Xu performed the materials preparation, materials characterization, and performance evaluation. Chen Hou performed the STEM‐EDS tests. Jiawei Hu, Hao Xu, Jiawei Zhong, and Chuande Huang analyzed the data. Jiawei Hu and Hao Xu wrote the original manuscript. All authors discussed the results and revised the manuscript. Jiawei Hu and Wei Wei supervised the project.

## Conflicts of Interest

The authors declare no conflict of interest.

## Supporting information




**Supporting File**: advs75455‐sup‐0001‐SuppMat.docx.

## Data Availability

The data that support the findings of this study are available from the corresponding author upon reasonable request.
