# Supplementary material for: Reaction‐Induced Reversible Reconstruction Enhanced Ni‐MgO/CaO Dual Functional Material for Stable CO2 Capture and In Situ Conversion
Source: Adv Sci (Weinh). 2026 Apr 27;13(41):e75455. doi: 10.1002/advs.75455 (PMC13335702; doi:10.1002/advs.75455)
Supplement: Supplementary file 1 — Supporting File: advs75455‐sup‐0001‐SuppMat.docx. [file ADVS-13-e75455-s001.docx]

Supporting Information

Reaction-Induced Reversible Reconstruction Enhanced Ni-MgO/CaO Dual Functional Material for Stable CO_2_ Capture and In-Situ Conversion

*Hao Xu*,^[a,e]^ *Chen Hou*,^[b]^ *Jiawei Zhong*,^[c]^ *Chuande Huang*,^[d,e]^ *Wei Wei**^[a,e]^ and *Jiawei Hu**^[a,e]^

[a] H. Xu, W. Wei, J. Hu
Center for Low-Carbon Conversion Science and Engineering; State Key Laboratory of Low Carbon Catalysis and Carbon Dioxide Utilization
Shanghai Advanced Research Institute, Chinese Academy of Sciences
Shanghai 201210, China
E-mail: weiwei@sari.ac.cn, hujw@sari.ac.cn

[b] C. Hou
Shanghai Synchrotron Radiation Facility
Shanghai Advanced Research Institute, Chinese Academy of Sciences
Shanghai 201204, China

[c] J. Zhong
Institute of Biomass Engineering
South China Agricultural University
Guangzhou 510642, China

[d] C. Huang
CAS Key Laboratory of Science and Technology on Applied Catalysis
Dalian Institute of Chemical Physics, Chinese Academy of Sciences
Dalian 116023, China

[e] H. Xu, C. Huang, W. Wei, J. Hu
University of Chinese Academy of Sciences
Beijing 100049, China

**Table of Contents**

Experimental Section…………………………………………………………………………………………………………3

Supplementary Figures.............................................................................................................................................6

Supplementary Tables............................................................................................................................................34

Reversible Reconstruction Kinetics Study..............................................................................................................41

Supplementary References....................................................................................................................................45

**Experimental Section**

**Materials preparation**

***Chemicals:*** Calcium acetate monohydrate (Ca(CH_3_COO)_2_·H_2_O, ≥ 99.0%), magnesium acetate tetrahydrate (Mg(CH_3_COO)_2_·4H_2_O, 99.9% metals basis), polyvinylpyrrolidone (PVP_10_, average molecular weight = 58000), citric acid (C_6_H_8_O_7_, ≥ 99.5%), urea (CO(NH_2_)_2_, 99%), ethylene glycol (C_2_H_6_O_2_, ≥ 99.5%), anhydrous ethanol (99.7%), and nickel acetylacetonate (Ni(acac)_2_, 95% metals basis) were purchased from Macklin. All chemicals were used as received. Deionized H_2_O was produced by a laboratory water purification system (HHitech).

***Support:*** A previous developed CO_2_ sorbent, i.e., hierarchically porous CaO incorporating highly dispersed MgO (labeled as CaMg), was employed as the support for dual-functional materials. Typically, 2.64 g of Ca(CH_3_COO)_2_·H_2_O, 3.22 g of Mg(CH_3_COO)_2_·4H_2_O, 16.67 g of PVP_10_, 5.76 g of C_6_H_8_O_7_, 3.4 mL of C_2_H_6_O_2_, 4.5 g of CO(NH_2_)_2_ and 120 mL of deionized H_2_O were thoroughly stirred at room temperature for 2 hours to form a transparent solution. The solution was then transferred into a 200 mL Teflon-lined stainless-steel autoclave and stood at 120°C for 24 hours in an oven. After naturally cooling down, the yielded precipitate was collected and thoroughly washed by water and ethanol with assistance of sonication, followed by drying at 60°C overnight. Subsequently, the dried solid was grinded and subjected to a temperature-programmed calcination (at 160°C for 30 minutes, then at 320°C for 30 minutes, and then at 500°C for 30 minutes, finally to 700°C; the temperature ramping rate maintained at 2 °C min^-1^) under air in a Muffle furnace to obtain the CaMg material. More explanation for this synthesis method can be found elsewhere.^[1]^

***Dual functional materials:*** The Ni-MgO/CaO dual functional materials (DFMs) were prepared via the designed method comprising an organic solution-assisted impregnation followed by a two-step calcination in different atmospheres. Specifically, the required amount (based on the predetermined Ni loading, i.e., 5, 10, 15, 20 wt.%) of Ni(acac)_2_, 1 g of CaMg and 50 mL of anhydrous ethanol were thoroughly mixed under sonication for at least 1 hour until the nickel precursor was completely dissolved. The resultant suspension was then treated by rotary evaporation (at 50°C and 100 mbar for 1 hour) to remove ethanol, followed by fully drying at 60°C overnight in an oven. Subsequently, the dried sample was ground and calcined in a tube furnace to yield the DFM. The first-step calcination was conducted at 360°C (heating rate: 1 °C min^-1^) for 2 hours in N_2_ flow. After naturally cooling down, the sample was washed for several times using anhydrous ethanol, and dried at 60°C overnight. Afterwards, the sample underwent the second-step calcination at 650°C (heating rate: 1 °C min^-1^) for 5 hours in pure air.

**Materials characterization**

***Inductively Coupled Plasma Optical Emission Spectroscopy (ICP-OES):*** The actual contents of Ni, Ca and Mg in the as-prepared DFMs were determined by ICP-OES (Agilent 5110 model instrument). Sample was prepared by dissolving around 20 mg of the DFM with 5 mL of aqua regia (HCl: HNO_3_ = 3:1), followed by dilution with deionized H_2_O to yield a volume of 25 mL.

***N_2_ Physisorption:*** The texture properties (i.e., specific surface area, pore size distribution and pore volume) of the materials were measured by N_2_ physisorption at -196°C (BSD-660M physical adsorption analyzer). Prior to measurements, the samples were in-situ degassed at 300°C for 6 hours. The specific surface area was calculated by Brunauer-Emmett-Teller (BET) method using the data in the p/p_0_ ranging from 0.06 to 0.30 on adsorption isotherm. The pore size distribution and pore volume were determined by fitting the desorption isotherm based on Barrett-Joyner-Halenda (BJH) theory.

***X-Ray Diffraction (XRD):*** The crystal composition of the materials in different states was characterized by XRD (Rigaku, Ultima IV X-ray diffractometer with Cu Kα radiation, operated at 40 mA and 40 kV). XRD patterns were collected in 2θ range of 10° to 90° with a step size of 0.02° and a scan speed of 2° min^-1^. Diffraction peaks of the known crystal phases were identified by referencing their corresponding number in the Powder Diffraction File (PDF) database. The crystallite size (d_XRD_, nm) of the target phase was estimated using the Scherrer’s equation:^[2]^

$d_{\mathrm{XRD}}=\frac{K\lambda}{\beta\cos\theta}$ (1)

where K is the shape constant (0.9 for spherical particle model), λ is the incoming X-ray wavelength (0.154 nm for Cu Kα radiation), β is the full width at half maximum intensity (FWHM), and θ is the Bragg angle (i.e., peak position) of the corresponding diffraction, both obtained by Gaussian fitting.

***X-ray Photoelectron Spectroscopy (XPS):*** The surface species of the DFMs in different states were determined by X-ray photoelectron spectroscopy (XPS), performed on a Thermo Scientific K-Alpha spectrometer with an Al K-a ray light source (12 kV, 4 mA, *hv* = 1486.6 eV) under ultrahigh vacuum. The obtained XPS spectra were analyzed using Avantage software. All binding energies were calibrated by setting a reference signal of C 1s peak at 284.8 eV. The concentration of the surface species was calculated by:

$C_{i}=\frac{A_{i}}{A}\times100$ (2)

where C_i_ (%) and A_i_ are the concentration and the fitted peak area of the surface species (i), respectively. A is the total area of the fitted peaks corresponding to the element.

***Electron Microscopy:*** The morphology of the materials in different states was observed by field-emission scanning electron microscope (FESEM) on ZEISS Gemini 300 (operated at 10 kV, with SE2 detector), and the combined energy-dispersive X-ray spectroscopy (EDS) analysis was performed to determine the surface atomic concentrations. The specimen was prepared by evenly spreading the powdered sample on a carbon tape.

The nanostructure of the materials was characterized by high-angle annular dark-field scanning transmission electron microscopy (HAADF-STEM, JEOL JEM-F200 microscope) combined with energy-dispersive X-ray spectroscopy (EDS, dual JED-2300T detectors) operated at 200 kV. High resolution TEM images were acquired with a Gatan Rio 1618 camera. The samples were ultrasonically dispersed in ethanol and then a drop of the solution was deposited onto a duplex copper grid coated with thin lacey carbon films.

***Raman Spectroscopy:*** The qualitative analysis of the deposited carbon was carried out using Raman spectroscopy on a Renishaw inVia Reflex with 532 nm laser excitation. Before testing, the powdered samples were compressed into thin sheet and placed on a microscope slide. The samples were scanned from 1000 cm^-1^ to 4000 cm^-1^ and each sample was tested at least 3 times at different positions to check for spectral reproducibility.

***Fourier Transform Infrared Spectroscopy (FTIR):*** The functional groups and chemical bonds on the materials from different stages of the designed preparation method were determined by FTIR (on a Thermo Scientific Nicolet iS20 spectrometer). All measurements were carried out under ambient atmosphere using the KBr pellet technique. The background spectrum was collected using pure KBr, and the samples were prepared by mixing the material with KBr at a mass ratio of 1:200. Each spectrum was acquired with 32 scans at a spectral resolution of 4 cm^-1^, over a wavenumber range of 500 ~ 2500 cm^-1^.

***Thermogravimetric Analysis (TGA):*** The amount of the deposited carbon on the spent materials was determined by TGA (on TA Q50 instrument). Typically, about 10 mg of sample was uniformly placed in a 100 μL platinum crucible. During test, the samples were heated from room temperature to 800°C (heating rate: 10°C min^-1^) in a pure air flow (100 mL min^-1^), and the weight variation was online recorded.

The isothermal CO_2_ adsorption and desorption performance of the materials was also investigated by TGA. The sample (approximately 10 mg) was pre-calcined at 650°C (heating rate: 10 °C min^-1^) for 30 minutes in N_2_ to ensure that the carbonation reaction started from the CaO phase. After cooling to 620°C in N_2_, 15 cycles of CO_2_ adsorption and desorption proceeded while the weight variation was online recorded. Each cycle comprises 40-minute adsorption step in 5 vol.% CO_2_/N_2_ followed by 40-minute desorption step in pure N_2_. The gas flow rate was fixed at 100 mL min^-1^. The weight gain during the adsorption step and the weight loss during the desorption step originate from CO_2_ capture and release, respectively. The CO_2_ uptake (mmol g^-1^) was calculated based on:

$CO_{2}uptake=\frac{\Delta m_{\mathrm{adsorption}}\times1000}{m_{pre-calcined}\times44.01}$ (3)

where $\Delta m_{\mathrm{adsorption}}$ (g) is the weight gain during the adsorption step, and $m_{pre-calcined}$ (g) is the weight recorded after pre-calcination step.

***Chemisorption Analysis:*** The reducibility, surface alkalinity and Ni dispersion of the materials were respectively studied via H_2_ temperature-programmed reduction (H_2_-TPR), CO_2_ temperature-programmed desorption (CO_2_-TPD) and H_2_ pulse chemisorption, using an automatic chemisorption analyzer (BSD-Chem C200) equipped with a thermal conductivity detector (TCD). The sample mass was 30 mg. The flow rate of feed gases was fixed at 30 mL min^-1^, and the temperature ramping rate was 10 °C min^-1^. **For H_2_-TPR**, the sample was pretreated at 700°C for 30 minutes in Ar flow to completely remove the moisture and carbonate species. After cooling to 50°C, the feed gas was switched to 10 vol.% H_2_/Ar and kept until the TCD signal was stable. Subsequently, the sample was heated from 50°C to 950°C while the consumption of H_2_ was online recorded. **For CO_2_-TPD**, the sample was in-situ reduced at 650°C for 30 minutes in 10 vol.% H_2_/Ar, and then purged by Ar at 650°C for additional 30 minutes. After cooling to 50°C in Ar, the feed gas was switched to 10 vol.% CO_2_/Ar for CO_2_ adsorption. After 1 hour, the feed gas was switched to He and maintained for 30 minutes to remove the residual gaseous CO_2_. Finally, the sample was heated from 50°C to 950°C in He while the desorption of CO_2_ was online recorded. **For H_2_ pulse chemisorption**, the sample was in-situ reduced by 10 vol.% H_2_/Ar at 650°C for 30 minutes, and subsequently purged by Ar at 650°C for 1 hour to completely remove the gaseous and adsorbed H_2_. After cooling to 50°C, H_2_ chemisorption was performed by repeatedly pulsing 10 vol.% H_2_/Ar (0.023 mL per pulse) with 10 mL min^-1^ Ar as carrier gas until adsorption saturation, consequently the total H_2_ adsorption capacity (Q, mL) was obtained. The Ni dispersion (D_Ni_, %) was calculated based on the following equation:

$D_{\mathrm{Ni}}=\frac{2\times Z\times Q/224}{m\times w/M}$ (4)

where Z is the stoichiometry factor of the dissociative H atom on Ni atom (= 1),^[3-4]^ and m, w and M respectively represent the sample mass (g), the loading amount of Ni (wt.%) and the molar weight of Ni (g mol^-1^).

**Performance Evaluation**

The performance of the as-prepared DFMs on CO_2_ capture and in-situ reforming of CH_4_ was thoroughly assessed in a fixed-bed quartz reactor (inner diameter: 8 mm) at atmospheric pressure (0.1 MPa). Typically, 0.1 g of the material was diluted with 1.4 g of inert quartz (80 ~ 120 mesh) and placed in the middle of the reactor (constant temperature zone). The flow rates of the inlet gases were controlled by the calibrated KOFLOC mass flow controllers, and the total flow rate of feed gases was fixed at 50 mL min ^1^. The outlet products were online monitored by either a gas chromatography (GC, using Ar as carrier gas) equipped with thermal conductivity detector (TCD) or a mass spectrometer (MS, TILON-LC-D200M). In MS spectra, H_2_, CH_4_, CO, Ar and CO_2_ were represented by the signals at m/z = 2, 16, 28, 40 and 44, respectively. Fragmentation patterns of the compounds were taken into account when assessing the product composition. The gas concentration in the outlet product was quantified based on standard gases, using internal standard method. Prior to performance evaluation, the material was firstly reduced in 5 vol.% H_2_/Ar at 650°C (heating rate: 10°C min^-1^) for 30 minutes. Four types of experiments were conducted, described as follows:

***CO_2_ Temperature-Programmed Capture (CO_2_-TPC):*** The dynamic CO_2_ capture-release behavior on the reduced materials was explored by CO_2_-TPC. After reduction, the sample was cooled to room temperature. Then, the feed gas was switched to 5 vol.% CO_2_/Ar and stabilized for 20 minutes. Afterwards, the sample was heated to 800°C with a heating rate of 10 °C min^-1^, while the outlet gases were online analyzed by MS.

***CH_4_*** ***Temperature-Programmed Surface Reaction (CH_4_-TPSR):*** The activation of CH_4_ on the carbonated materials was studied by CH_4_-TPSR. After reduction, the sample was cooled to 620°C, followed by purging with Ar for 30 minutes. Subsequently, the feed gas was switched to 5 vol.% CO_2_/Ar and maintained at 620°C for 1 hour to ensure a complete carbonation. After cooling to room temperature, the feed gas was switched to 5 vol.% CH_4_/Ar and stabilized for 20 minutes. Finally, the sample was heated to 800°C with a heating rate of 10 °C min^-1^, while the outlet gases were online analyzed by MS.

***Steady-State Dry Reforming of Methane (DRM):*** The catalytic performance of the DFMs was evaluated by conducting steady-state DRM experiments. After reduction, the sample was purged by Ar at 620°C for 30 minutes, and then carbonated in 5 vol.% CO_2_/Ar for 40 minutes. After purging with Ar for 5 minutes, the steady-state experiment was initiated upon switching feed gas to a mixture of CO_2_ (5 vol.%), CH_4_ (5 vol.%) and Ar (90 vol.%), and proceeded at 620°C for 20 hours. The outlet product was online analyzed by GC. CO_2_ conversion (%), CH_4_ conversion (%) and carbon balance (%) were calculated based on the following equations:

$\mathrm{CO}_{2} conversion=\frac{F_{\mathrm{CO}_{2},in}\left( t \right)-F_{\mathrm{CO}_{2},out}\left( t \right)}{F_{\mathrm{CO}_{2},in}\left( t \right)}\times100$ (5)

$\mathrm{CH}_{4} conversion=\frac{F_{\mathrm{CH}_{4},in}\left( t \right)-F_{\mathrm{CH}_{4},out}\left( t \right)}{F_{\mathrm{CH}_{4},in}\left( t \right)}\times100$ (6)

$Carbon balance=\frac{F_{\mathrm{CO}_{2},out}\left( t \right)+F_{\mathrm{CH}_{4},out}\left( t \right)+F_{CO,out}\left( t \right)}{F_{\mathrm{CO}_{2},in}\left( t \right)+F_{\mathrm{CH}_{4},in}\left( t \right)}\times100$ (7)

where $F_{\mathrm{CO}_{2},in}\left( t \right)$ and $F_{\mathrm{CH}_{4},in}\left( t \right)$ represent the inlet flow rates (ml min^-1^) of CO_2_ and CH_4_, $F_{\mathrm{CO}_{2},out}\left( t \right)$, $F_{\mathrm{CH}_{4},out}\left( t \right)$ and $F_{CO,out}\left( t \right)$ represent the outlet flow rates (ml min^-1^) of CO_2_ ,CH_4_ and CO.

***CaLDRM Cycles:*** The performance of DFMs in long-term CaLDRM process was assessed at 620°C for 65 cycles. After reduction, the sample was purged by Ar until no H_2_ residue could be detected. Afterwards, the CaLDRM cycles were performed by alternately switching feed gas and the outlet product was online analyzed by MS. Each cycle comprises 32-minute CO_2_ capture step (in 5 vol.% CO_2_/Ar) and 24-minute CO_2_ conversion step (in 5 vol.% CH_4_/Ar), with 2-minute Ar purging in between. CO_2_ uptake (mmol g^-1^), CO_2_ in-situ conversion (%), CH_4_ conversion (%) and syngas yield (mmol g^-1^) were calculated based on the following equations:

$\mathrm{CO}_{2} uptake=\frac{\int\left[ F_{\mathrm{CO}_{2},in}\left( t_{1} \right)-F_{\mathrm{CO}_{2},out}\left( t_{1} \right)-0.5\times F_{CO,out}\left( t_{1} \right) \right]dt_{1}}{22.4\times m}$ (8)

$\mathrm{CO}_{2} in­situ conversion=\frac{\mathrm{CO}_{2} uptake-\int F_{\mathrm{CO}_{2},out}\left( t_{2} \right)dt_{2}/22.4/m}{\mathrm{CO}_{2}\mathrm{uptake}}\times100$ (9)

$\mathrm{CH}_{4} conversion=\frac{\int\left[ F_{\mathrm{CH}_{4},in}\left( t_{2} \right)-F_{\mathrm{CH}_{4},out}\left( t_{2} \right) \right]\mathrm{dt}_{2}}{\int F_{\mathrm{CH}_{4},in}\left( t_{2} \right)\mathrm{dt}_{2}}\times100$ (10)

$Syngas yield=\frac{\int F_{CO,out}\left( t_{2} \right)\mathrm{dt}_{2}+\int F_{H_{2},out}\left( t_{2} \right)\mathrm{dt}_{2}}{22.4\times m}$ (11)

where $F_{\mathrm{CO}_{2},in}\left( t_{1} \right)$, $F_{\mathrm{CO}_{2},out}\left( t_{1} \right)$ and $F_{CO,out}\left( t_{1} \right)$ are the inlet flow rate (ml min^-1^) of CO_2_ and the outlet flow rates (ml min^-1^) of CO_2_ and CO during the CO_2_ capture step (t_1_), respectively. $F_{\mathrm{CH}_{4},in}\left( t_{2} \right)$, $F_{\mathrm{CH}_{4},out}\left( t_{2} \right)$, $F_{\mathrm{CO}_{2},out}\left( t_{2} \right)$, $F_{CO,out}\left( t_{2} \right)$, and $F_{H_{2},out}\left( t_{2} \right)$ are the inlet flow rate (ml min^-1^) of CH_4_ and the outlet flow rates (ml min^-1^) of CH_4_, CO_2_, CO and H_2_ during the CO_2_ conversion step (t_2_), respectively. Here, m is the sample mass (g).

**Supplementary Figures**


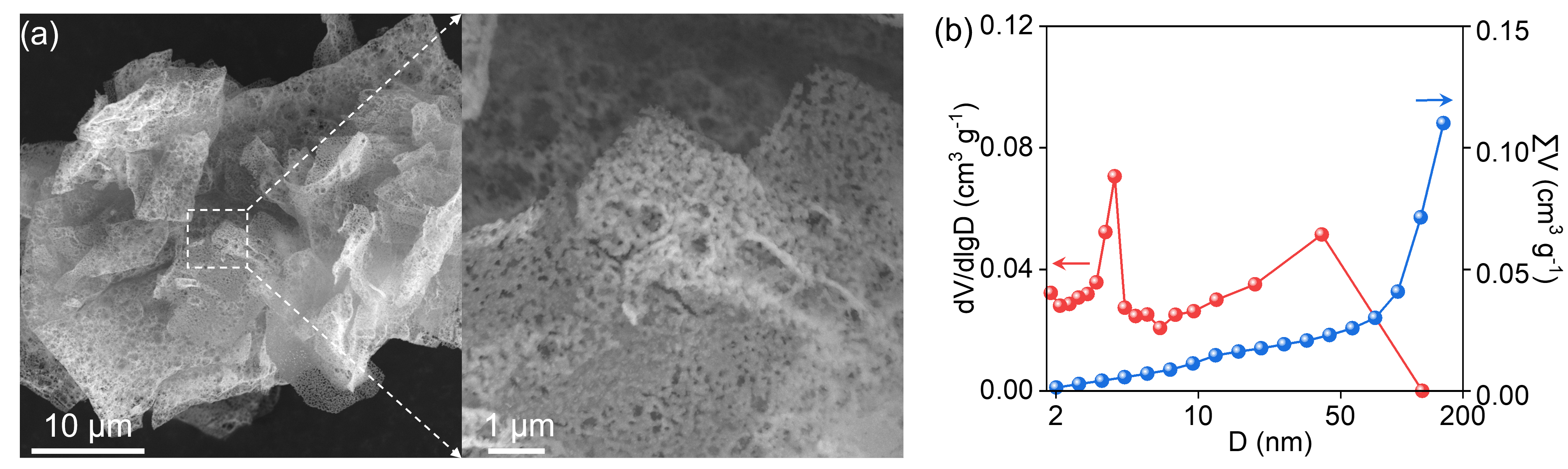


**Figure S1.** SEM images (a) and pore size distribution (b) of CaMg.


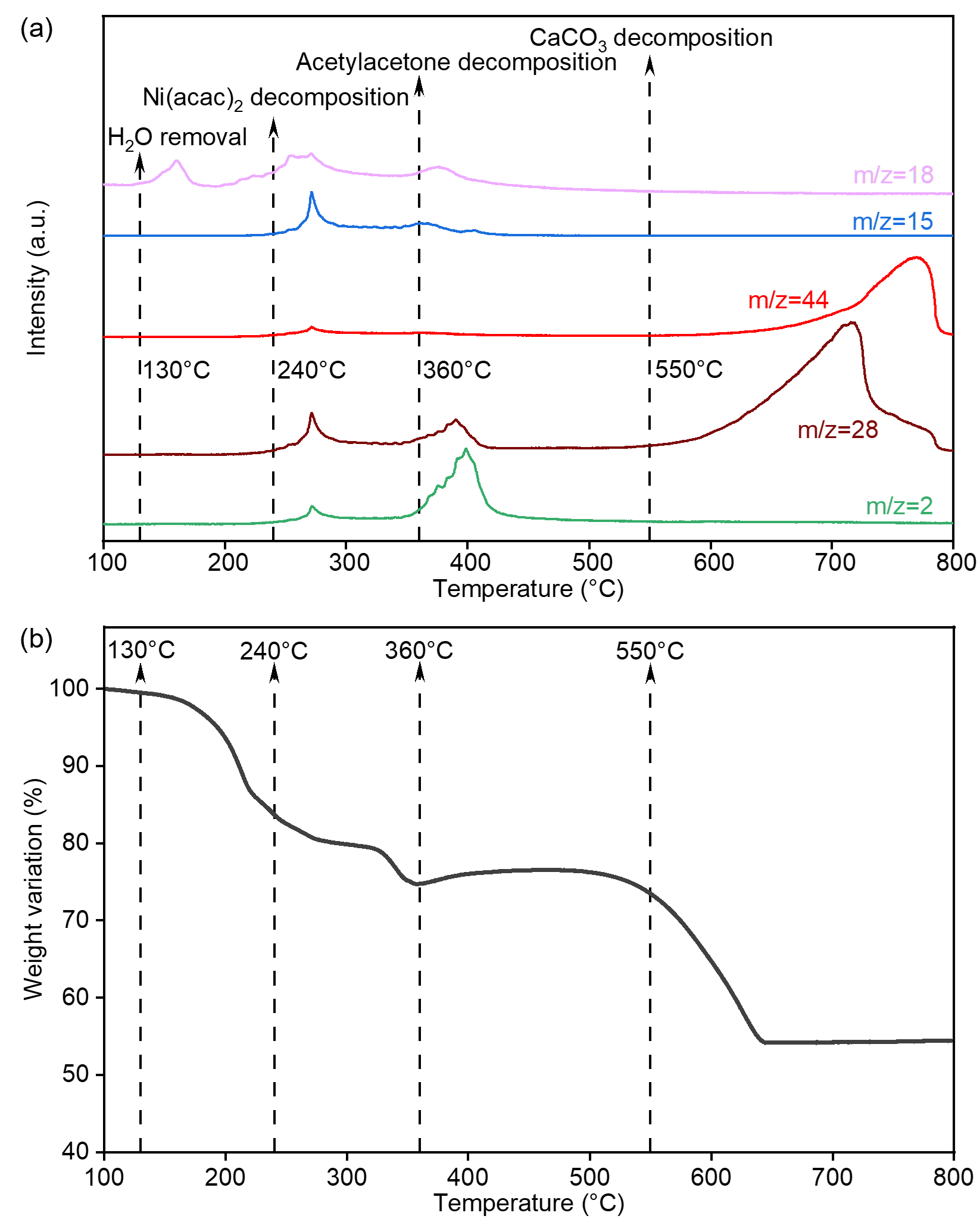


**Figure S2.** (a) Mass spectrum (MS) and (b) thermogravimetric (TG) profile recorded during the calcination of the freshly impregnated sample under Ar atmosphere; temperature ramping rate:10 °C min^-1^.

**Discussion:** Upon 130°C, MS shows a peak at m/z = 18, accompanied by a weight drop on TG profile, indicating the removal of adsorbed H_2_O. Within 240°C ~ 450°C, MS displays a series of peaks, assigned to H_2_O (m/z = 18), CO_2_ (m/z = 44), CO or the fragment of CO_2_ (m/z = 28), the fragment of acetylacetone (m/z = 15 and 2) and H_2_ (m/z = 2), indicating the gradual decomposition of Ni(acac)_2_, which result in a weight loss (from 240°C). Notably, the weight loss stops at 360°C, suggesting that the Ni(acac)_2_ has been completely decomposed at this temperature. Within 360°C ~ 450°C, the MS peaks represent the further decomposition of acetylacetone, since it is a gas phase reaction, which can not be detected by TG. Beyond 550°C, the significant weight drop originates from the decomposition of CaCO_3_, confirmed by the MS peaks of CO_2_ and CO.


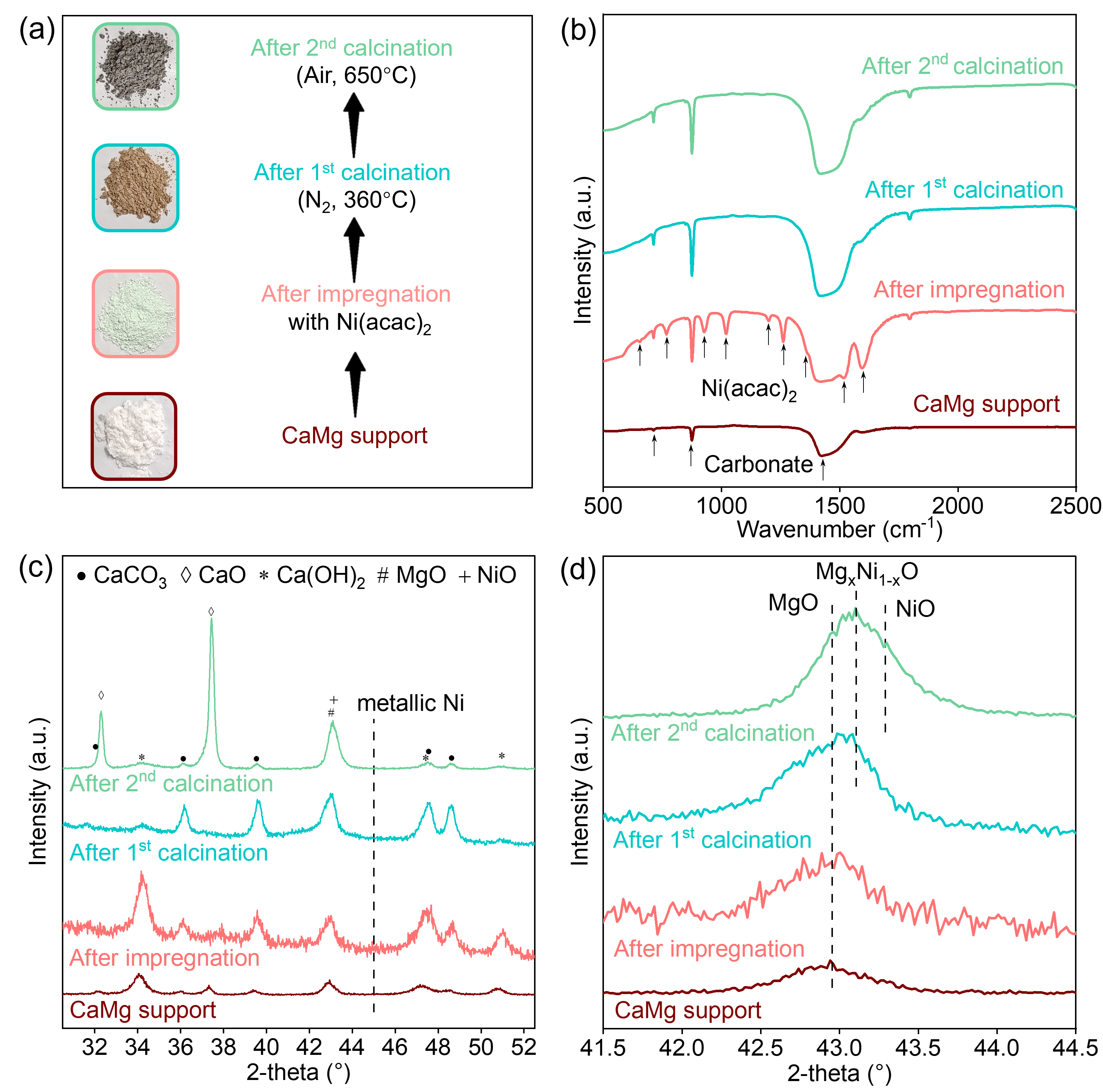


**Figure S3.** (a) Photographs, (b) FTIR spectra and (c) XRD patterns of the samples at different preparation stages; (d) a zoom of the XRD patterns (c) within the region of 41.5° ~ 44.5°.

**Discussion:** After impregnation, the sample color changed from white to light green (Figure S3a), and the FTIR spectrum showed the characteristic peaks of Ni(acac)_2_ (Figure S3b),^[5]^ confirming successful loading of the Ni precursor onto the CaMg support. Following the 1^st^ calcination step (in N_2_ at 360°C), all Ni(acac)_2_-related peaks disappeared, indicating its complete decomposition, consistent with the TGA results (Figure S2b). Notably, the FTIR spectrum after the 2^nd^ calcination step (in air at 650°C) remained unchanged from that after the 1^st^ step, suggesting that all organic species were already removed during the initial calcination. However, the sample color shifted from khaki to silver black (typically associated with supported NiO grains), implying that the Ni species existed in an intermediate state after the 1^st^ calcination step. XRD analysis revealed no new crystalline phases after impregnation relative to the support (Figure S3c), with a symmetric single peak observed at the MgO position (Figure S3d). After the 1^st^ calcination step, this peak became asymmetric and developed a minor shoulder at the position characteristic of the Mg_x_Ni_1-x_O solid solution, indicating the onset of Ni incorporation into the MgO lattice. Thermal decomposition of Ni(acac)_2_ under inert atmosphere (e.g., N_2_) is known to generate strongly reducing gases such as H_2_ and CO (Figure S2a), which can in situ reduce Ni species to the metallic state.^[6]^ Nevertheless, no metallic Ni phase was detected in the sample after the 1^st^ calcination (Figure S3c). Considering the complete decomposition of Ni(acac)_2_, it is deduced that the "Ni intermediates" correspond to highly dispersed Ni particles with a metallic surface, partially embedded within the MgO matrix. After the 2^nd^ calcination step, the diffraction peak attributed to the Mg_x_Ni_1-x_O solid solution became more pronounced, accompanied by a weakening of the MgO peak and the emergence of a NiO shoulder. These changes indicate complete oxidation of the Ni particles and the formation of a well-structured Mg_x_Ni_1-x_O solid solution.


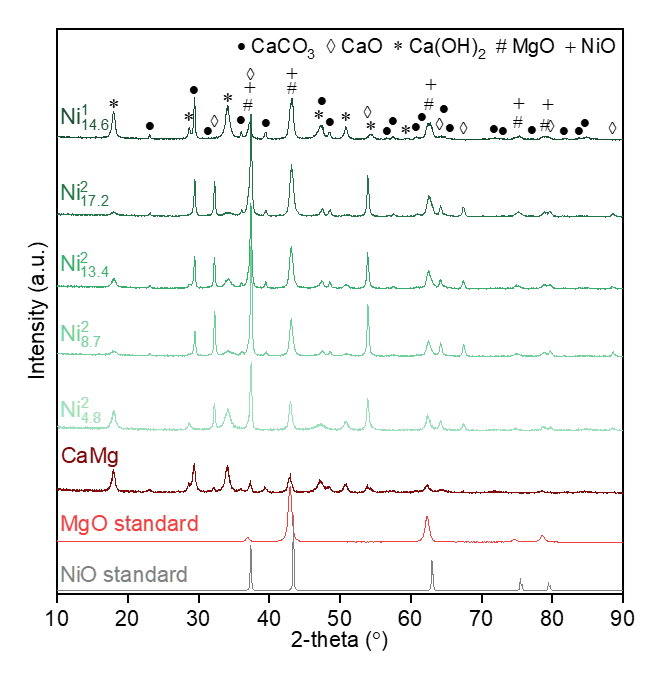


**Figure S4.** XRD patterns of the as-prepared materials. The PDF card number of the crystal phase corresponds to: CaCO_3_ (#99-0022), CaO (#77-2010), Ca(OH)_2_ (#81-2040), MgO (#43-1022), NiO (#89-7131).


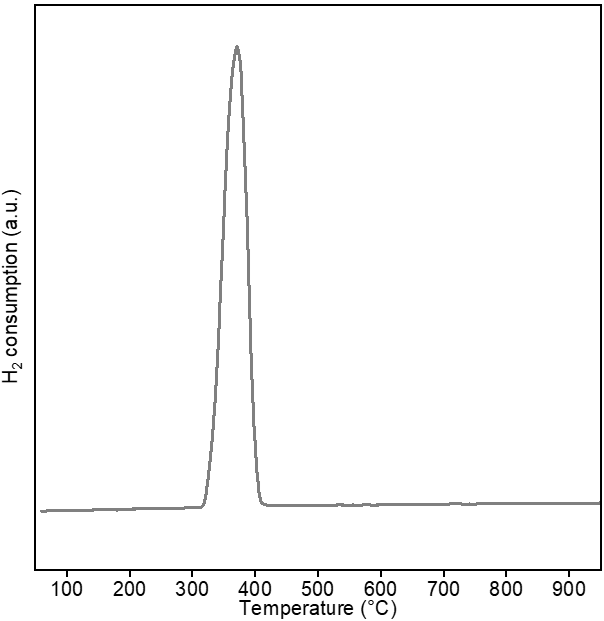


**Figure S5.** H_2_-TPR profiles of the NiO standard.


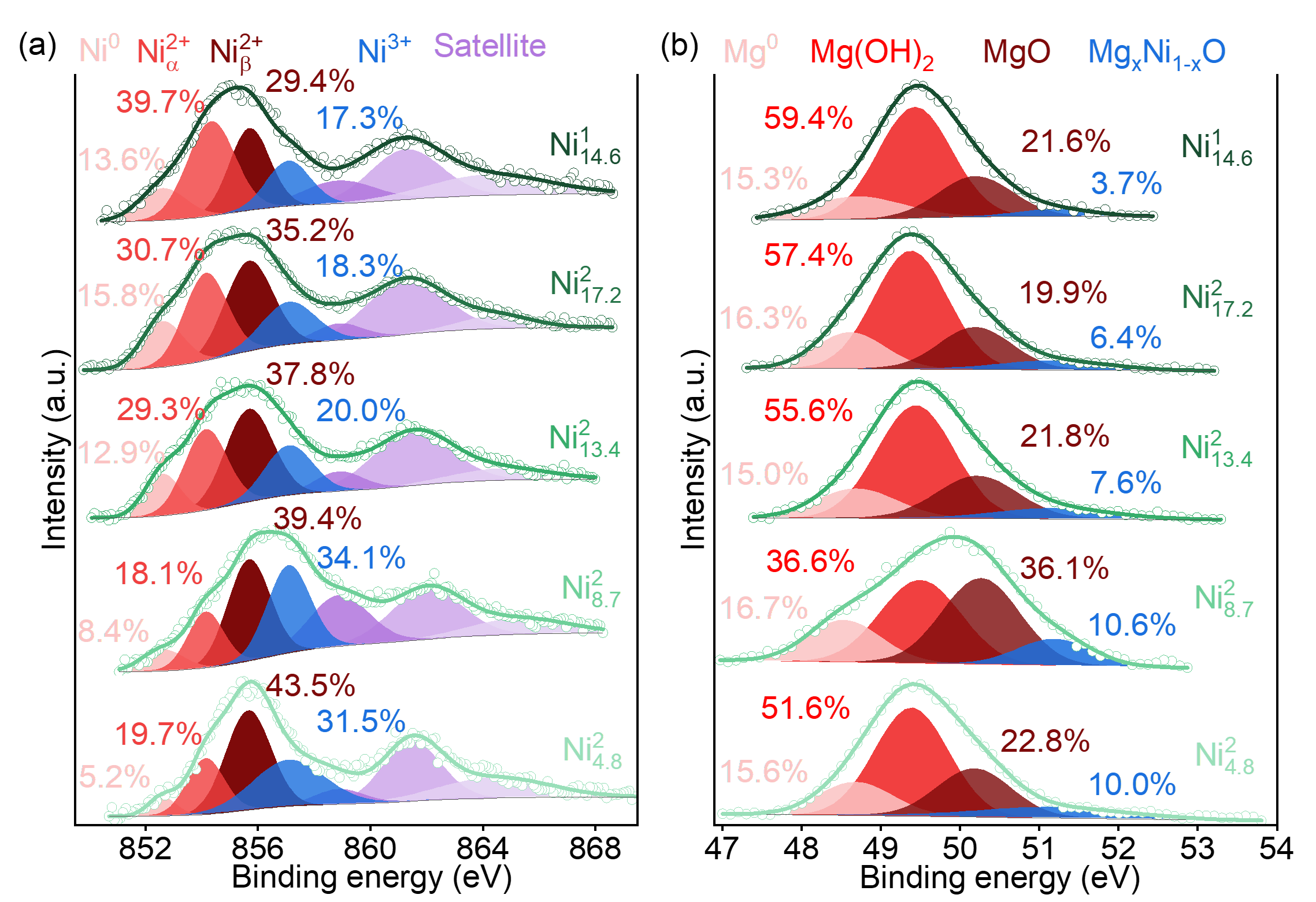


**Figure S6.** XPS spectra and the corresponding fitting curves of (a) Ni 2p and (b) Mg 2p in the reduced DFMs. The reduction treatment was conducted in 5 vol.% H_2_/Ar at 650°C (heating rate: 10 °C min^-1^) for 30 minutes.


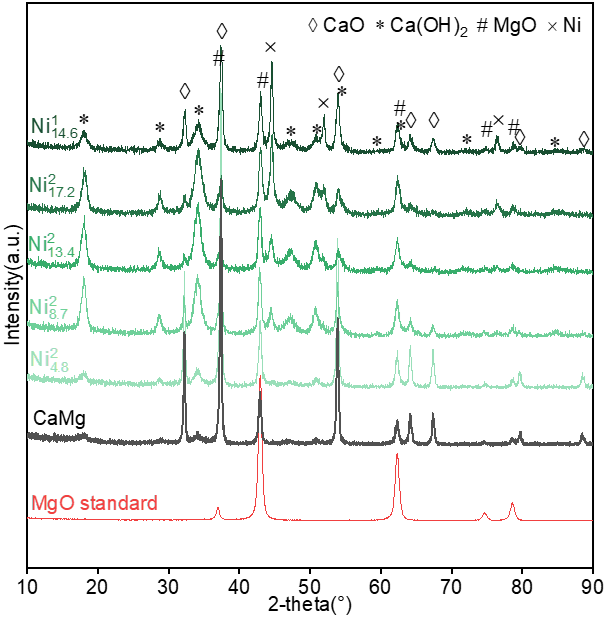


**Figure S7.** XRD patterns of the reduced materials. The PDF card number of the crystal phase corresponds to: CaO (#77-2010), Ca(OH)_2_ (#81-2040), MgO (#43-1022), Ni (#70-0989). The reduction treatment was conducted in 5 vol.% H_2_/Ar at 650°C (heating rate: 10 °C min^-1^) for 30 minutes.


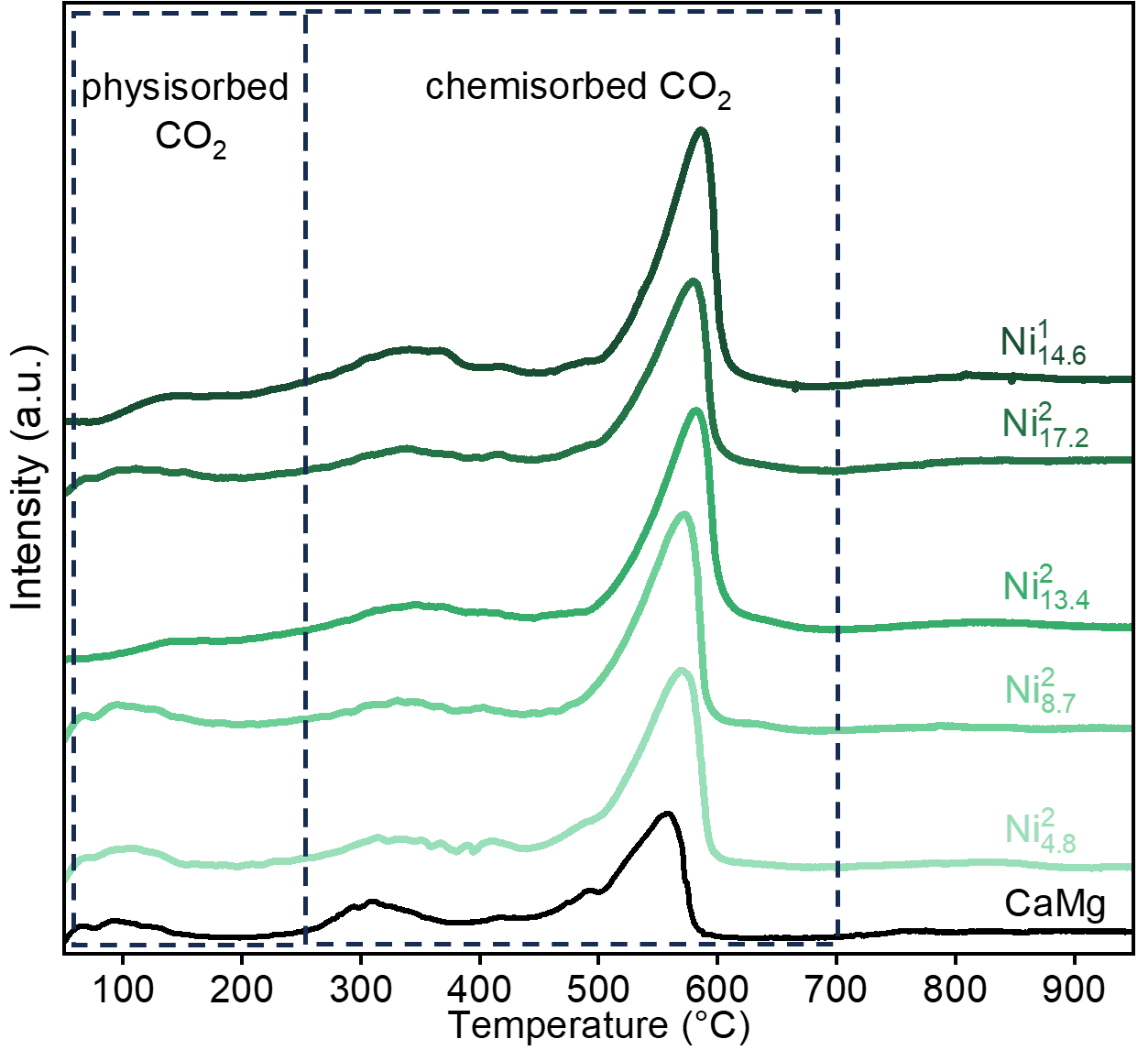


**Figure S8.** CO_2_-TPD profiles on the reduced materials.

**Discussion:** Two distinct peaks exist in the chemisorption region, which originate from the desorption of the CO_2_ adsorbed on weak (at 250 ~ 400°C) and strong alkaline (at 500 ~ 600°C) sites, respectively. As clearly seen, both peaks shift towards high temperature with loading Ni, indicating the enhancement of surface alkalinity.^[7]^ This is attributed to the increase of metallic Ni on the material surface (Figure S6), since CO_2_ can also be adsorbed and activated by Ni surface.^[8]^


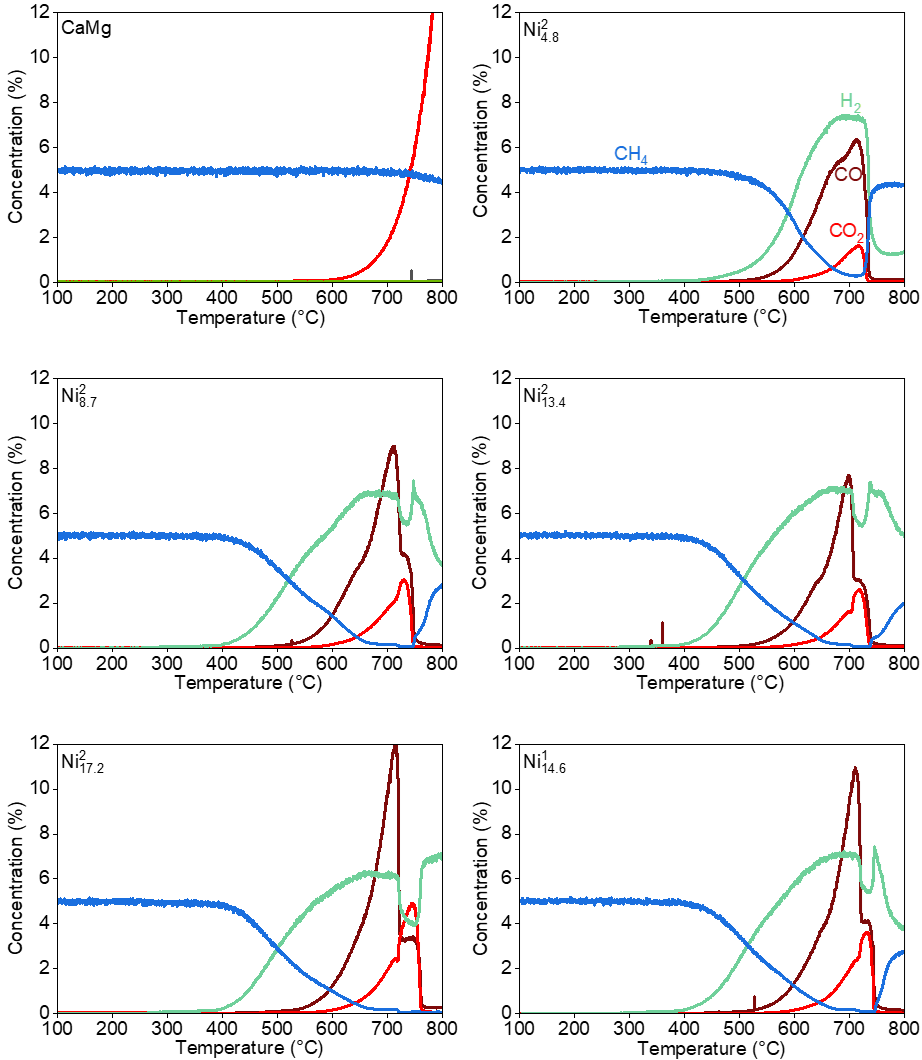


**Figure S9.** The evolution of outlet gas concentrations during CH_4_-TPSR experiments performed on different materials. Before TPSR, the materials were firstly reduced in 5 vol.% H_2_/Ar at 650°C for 30 minutes, followed by treated in 5 vol.% CO_2_/Ar at 620°C for 1 hour.


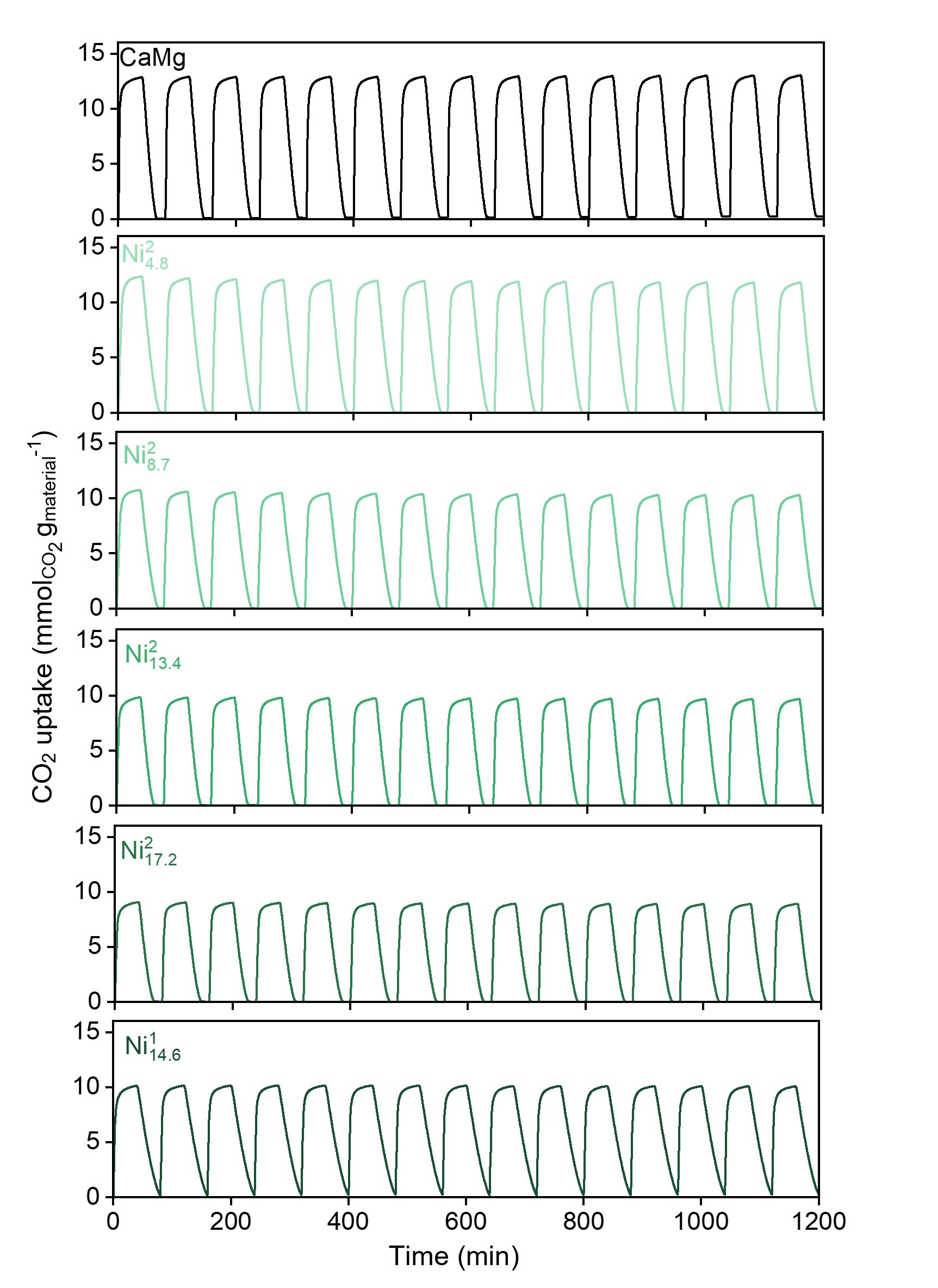


**Figure S10.** CO_2_ uptake recorded during isothermal adsorption-desorption cycles at 620°C performed on the as-prepared materials. One cycle comprises 40-minute adsorption step in 5 vol.% CO_2_/N_2_ followed by 40-minute desorption step in pure N_2_.


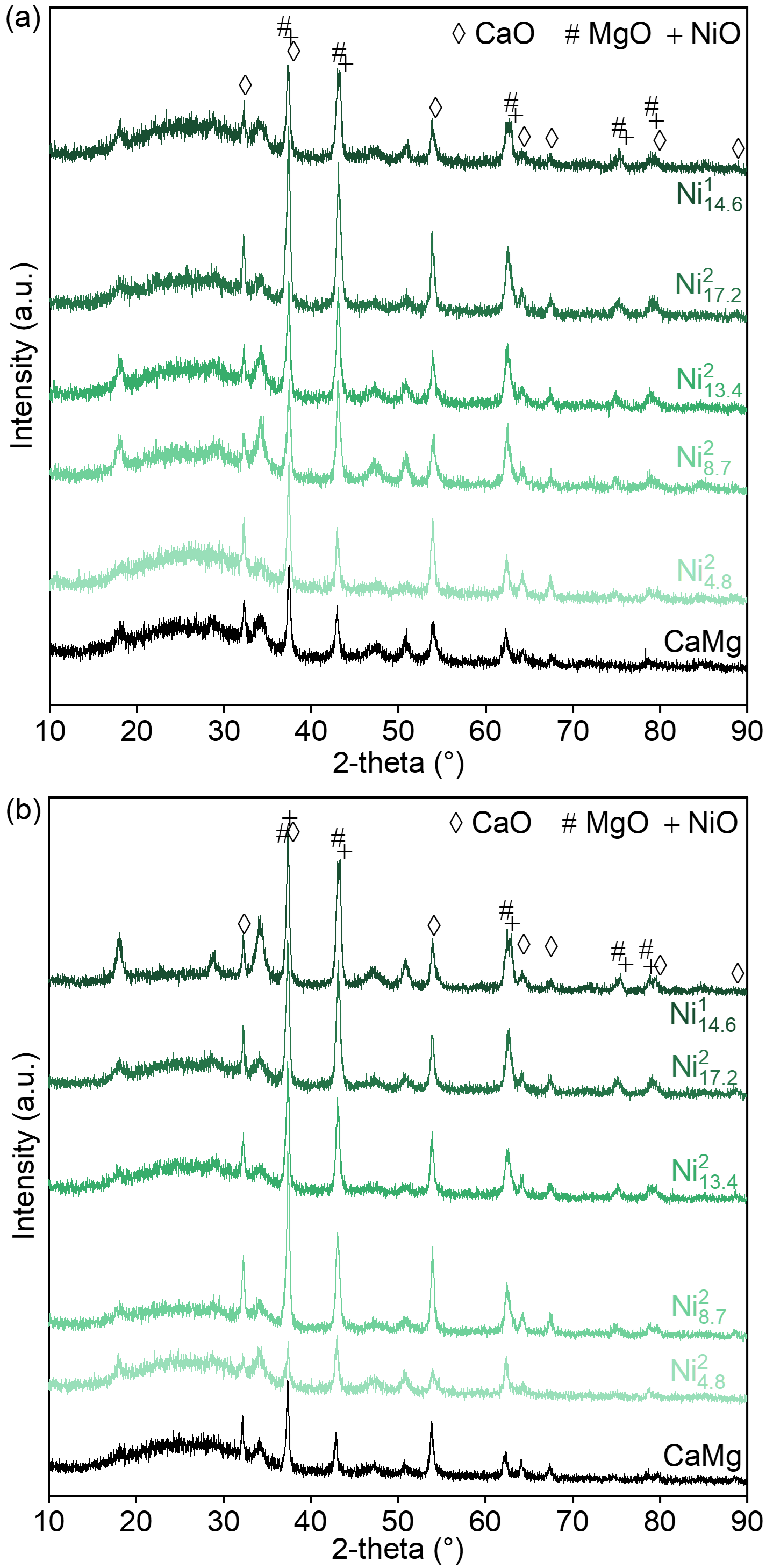


**Figure S11.** XRD patterns of the materials (a) before and (b) after 15 cycles of isothermal CO_2_ adsorption-desorption (Figure S10). The samples before cycles were obtained by calcination in TGA reactor at 650°C for 30 minutes under N_2_. The PDF card number of the crystal phase corresponds to: CaO (#77-2010), MgO (#43-1022), NiO (#89-7131). The unmarked diffraction peaks represent the Ca(OH)_2_ phase (PDF: #81-2040) derived by the strong hygroscopicity of CaO.


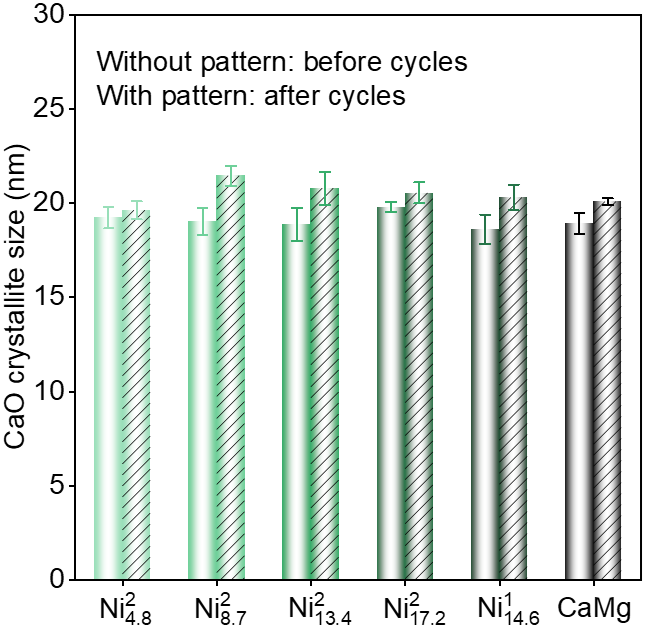


**Figure S12.** The crystallite size of CaO in the materials before and after 15 cycles of isothermal CO_2_ adsorption-desorption, calculated from the characteristic diffraction peaks (2θ = 32.19° and 53.84°) in XRD patterns (Figure S11) based on the Scherrer’s equation.


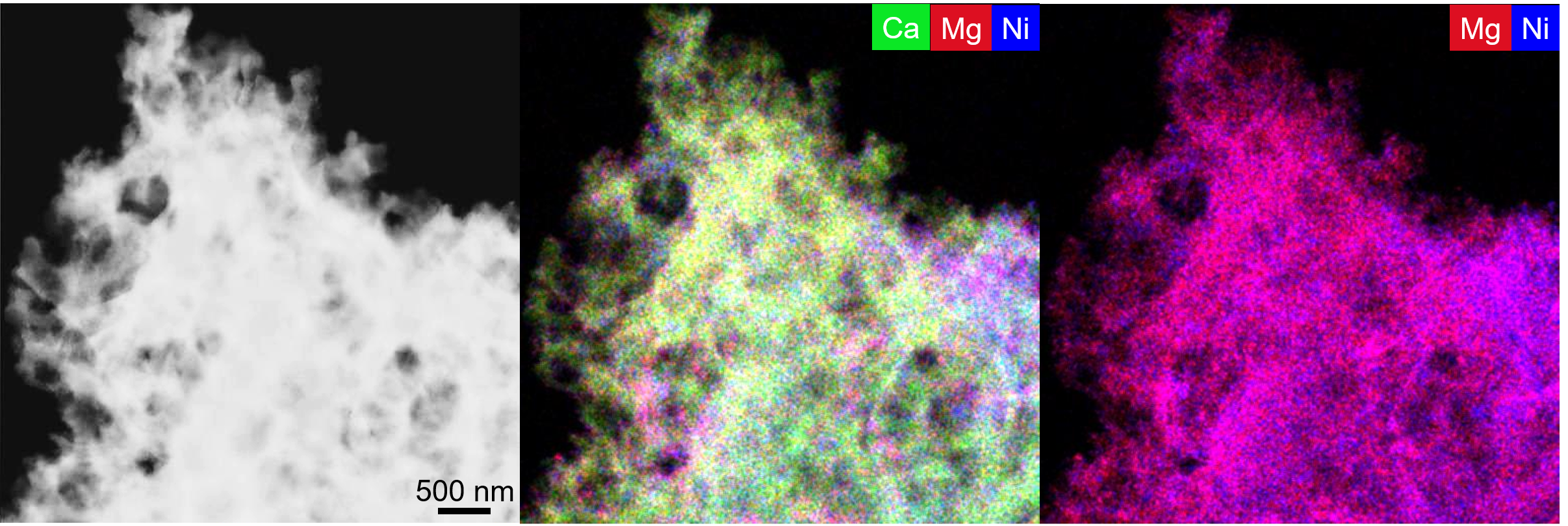


**Figure S13.** HAADF-STEM image and EDS elemental maps of the as-prepared Ni1 14.6.

**Figure S14.** CO_2_ conversion during 20-hour steady-state DRM experiments performed on the carbonated samples at 620°C; Equ._with or _without C: the thermodynamic equilibrium values calculated with or without considering the formation of carbon; mean values represent the average CO_2_ conversion during the 20-hour DRM.


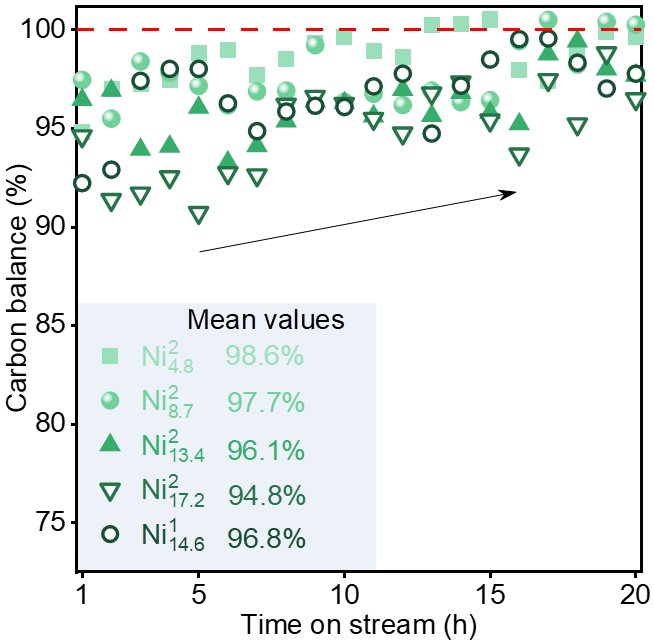


**Figure S15.** Carbon balance during 20-hour steady-state DRM experiments; mean values represent the average carbon balance during the 20-hour DRM.


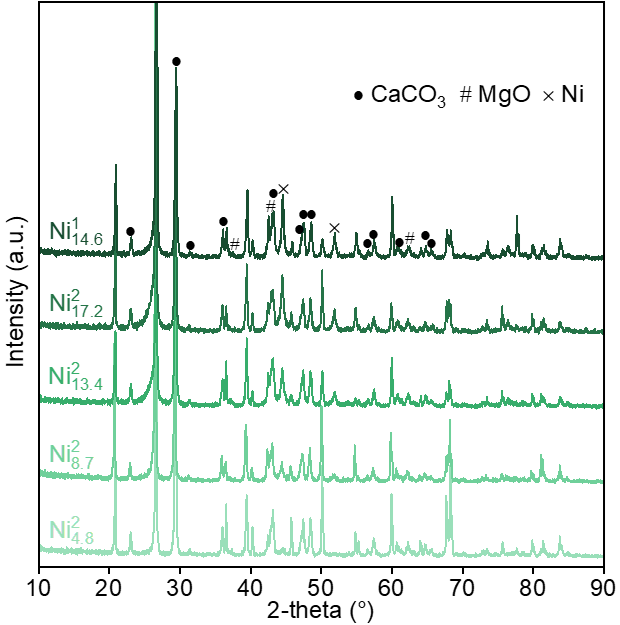


**Figure S16.** XRD patterns of the spent DFMs after 20-hour steady-state DRM reaction. The PDF card number of the crystal phase corresponds to: CaCO_3_ (#99-0022), MgO (#43-1022), Ni (#70-0989). The unmarked diffraction peaks originate from the quartz sand.


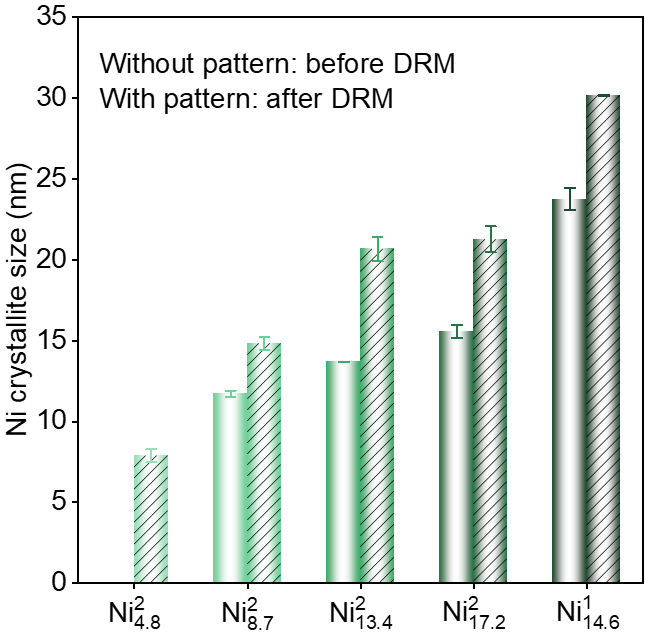


**Figure S17.** The crystallite size of Ni in the DFMs before (i.e., reduced sample) and after 20-hour steady-state DRM reaction, calculated from the characteristic diffraction peaks (2θ = 44.60° and 51.98°) in XRD patterns (Figures S7 and S16) based on the Scherrer’s equation. The crystallite size of Ni in the reduced Ni2 8.7 is not provided, because the metallic Ni phase is unobserved in the XRD pattern (Figure S7).


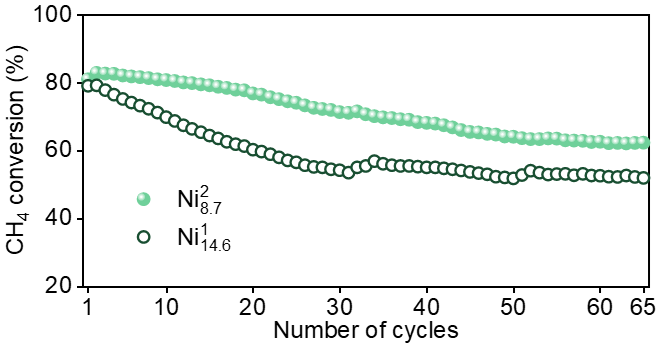


**Figure S18.** CH_4_ conversion during 65 cycles of CaLDRM implemented on the reduced Ni2 8.7 and Ni1 14.6 DFMs at 620°C.


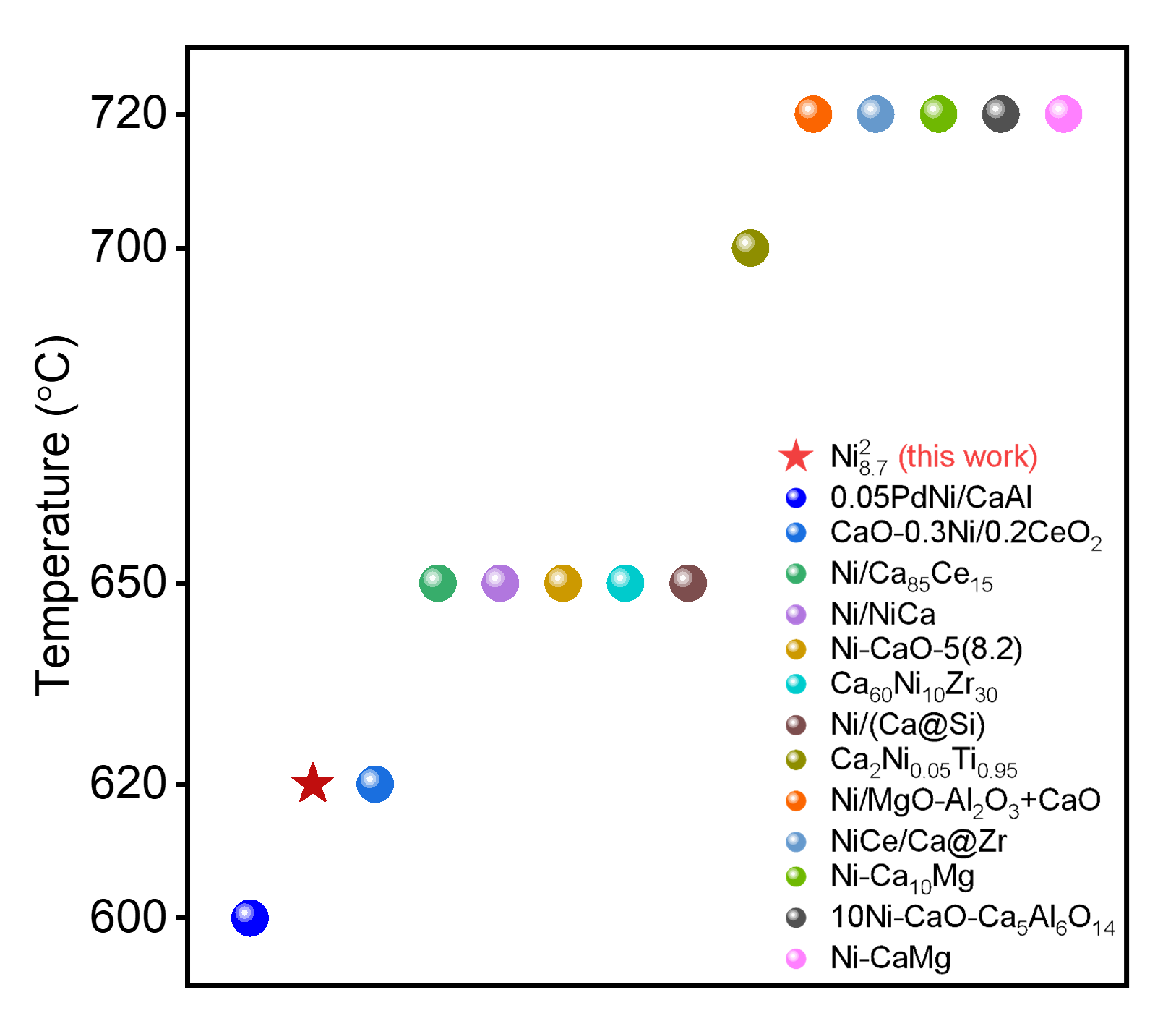


**Figure S19.** Comparison of the operating temperature of the CaLDRM cycles implemented on CaO-Ni based DFMs in the literature and this work.


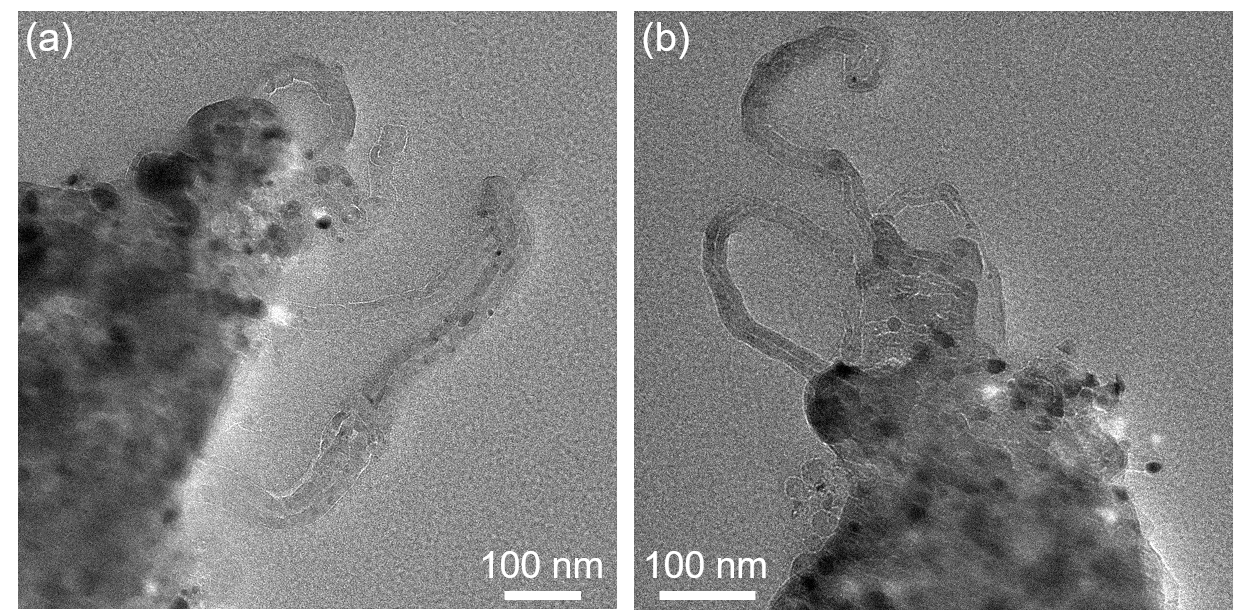


**Figure S20.** TEM images of (a) Ni2 8.7 and (b) Ni1 14.6 after 65 cycles of CaLDRM.


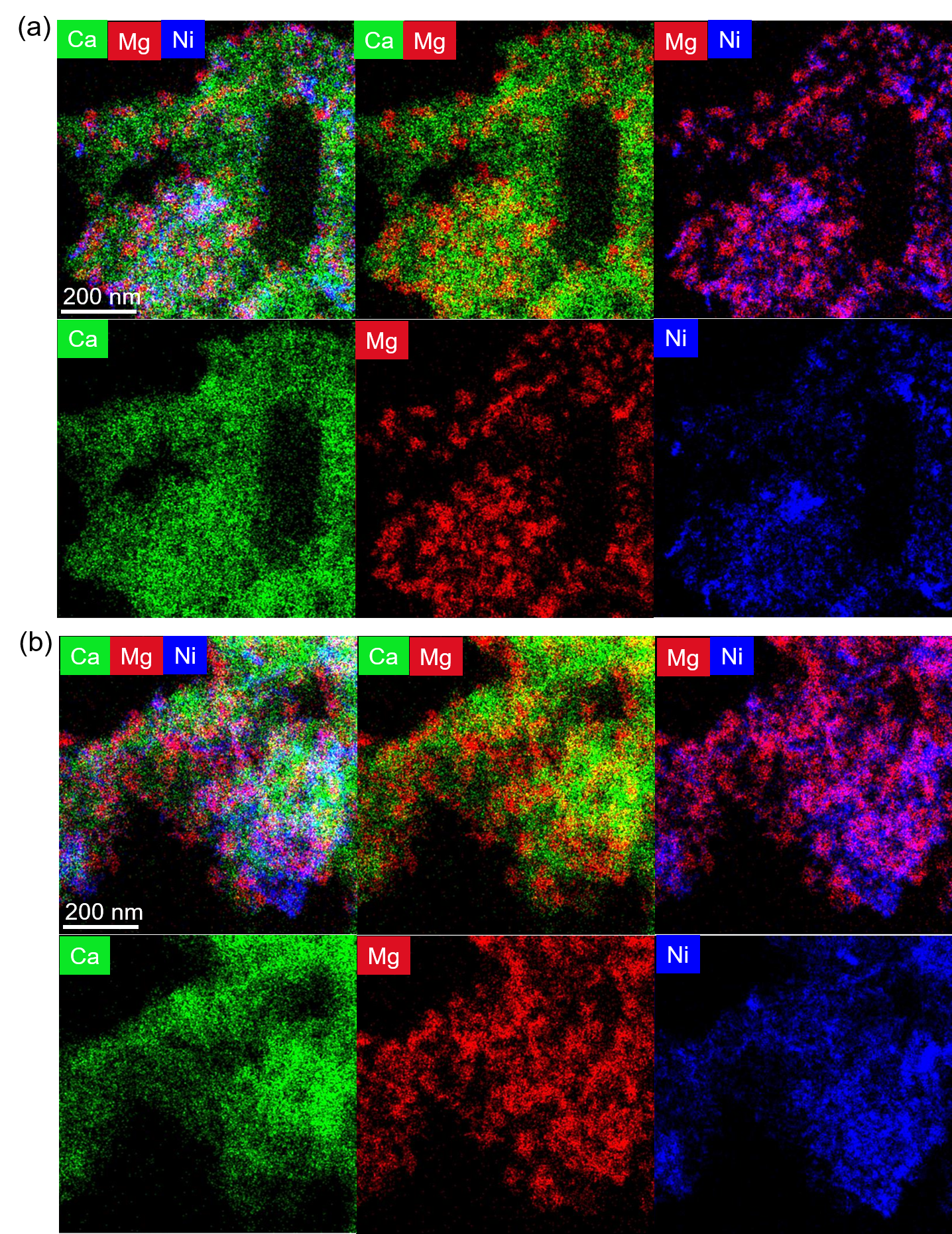


**Figure S21.** STEM-EDS elemental maps of (a) Ni2 8.7 and (b) Ni1 14.6 before (i.e., the reduced sample) 65 cycles of CaLDRM.


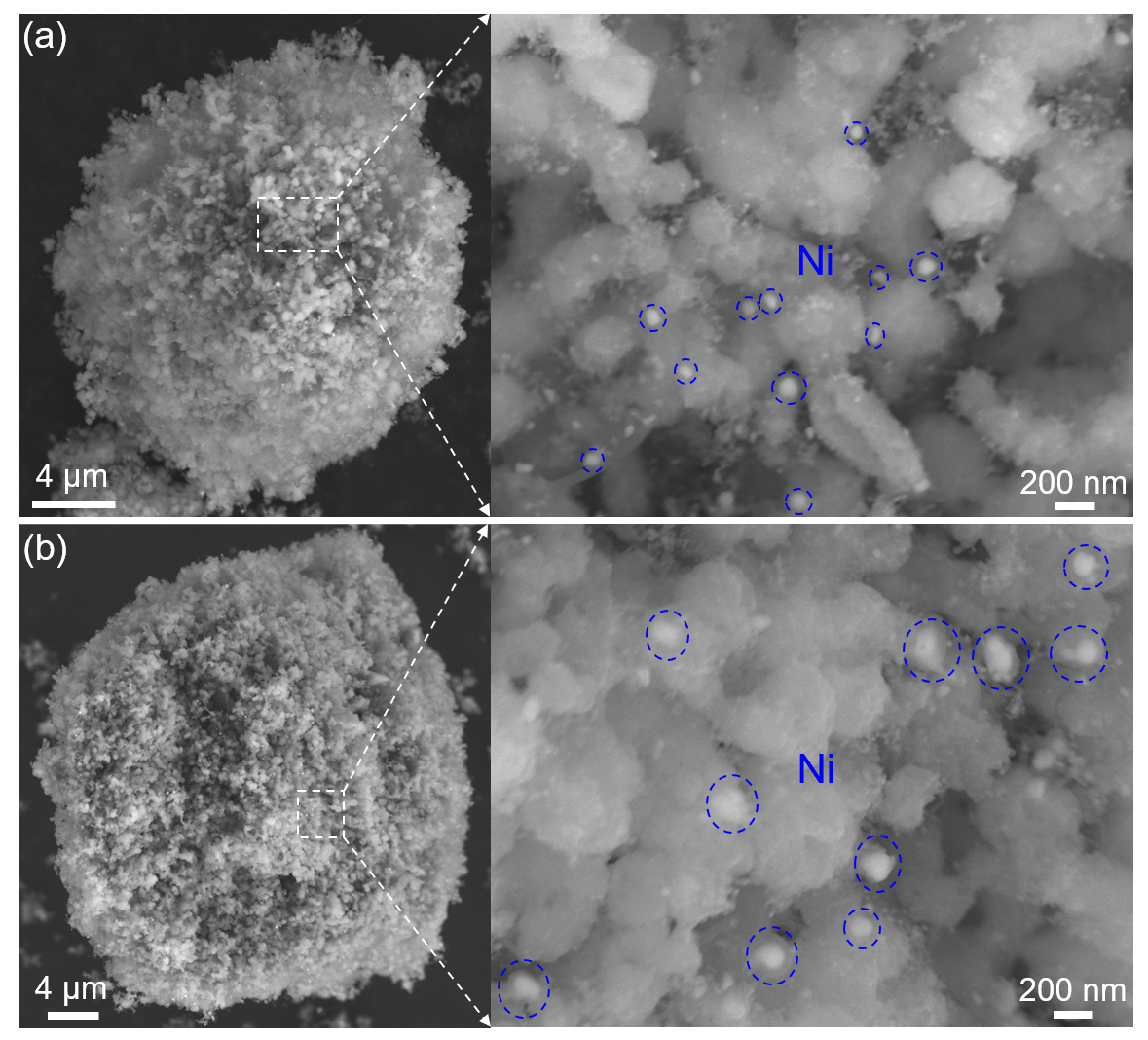


**Figure S22.** SEM images of the spent (a) Ni2 8.7 and (b) Ni1 14.6 after 65 cycles of CaLDRM.


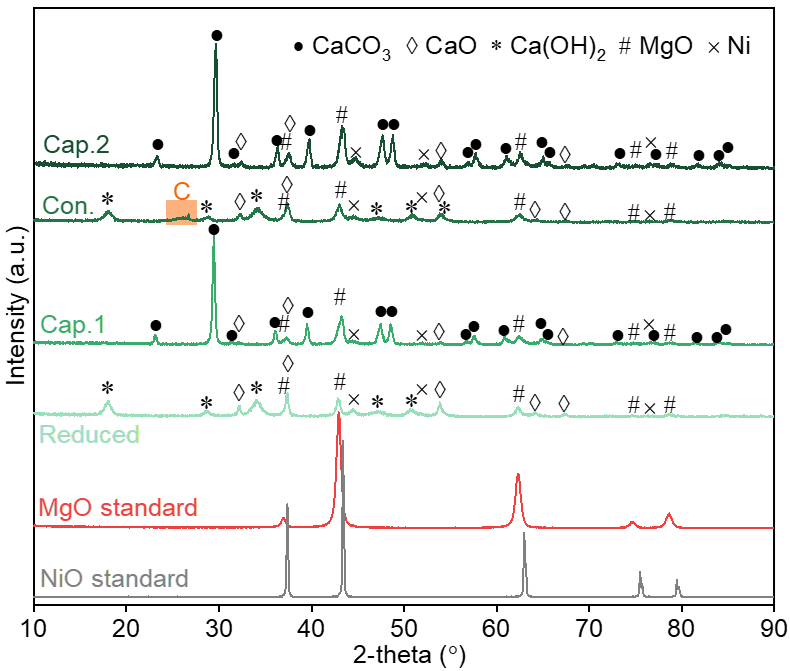


**Figure S23.** XRD patterns of the samples of Ni2 8.7 obtained from different steps in CaLDRM. Reduced, Cap.1, Con. and Cap.2 refer to the reduced sample, and the samples after the 1^st^ CO_2_ capture (in 5 vol.% CO_2_/Ar at 620°C for 32 minutes), the subsequent CO_2_ conversion (in 5 vol.% CH_4_/Ar at 620°C for 24 minutes) and the 2^nd^ CO_2_ capture steps (in 5 vol.% CO_2_/Ar at 620°C for 32 minutes), respectively. The PDF card number of the crystal phase corresponds to: CaCO_3_ (#99-0022), CaO (#77-2010), Ca(OH)_2_ (#81-2040), MgO (#43-1022), Ni (#70-0989).


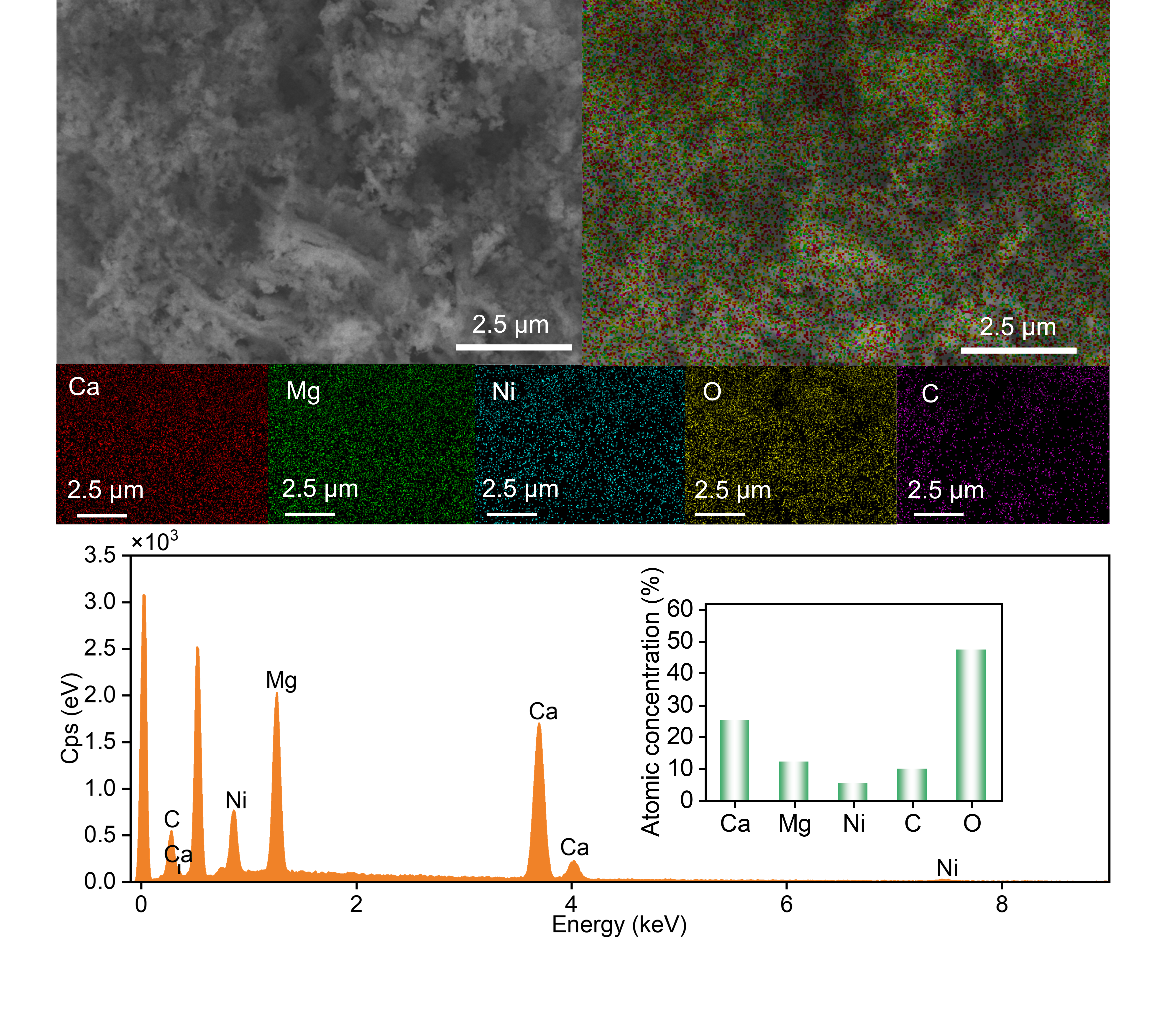


**Figure S24.** SEM-EDS analysis of the reduced Ni2 8.7.


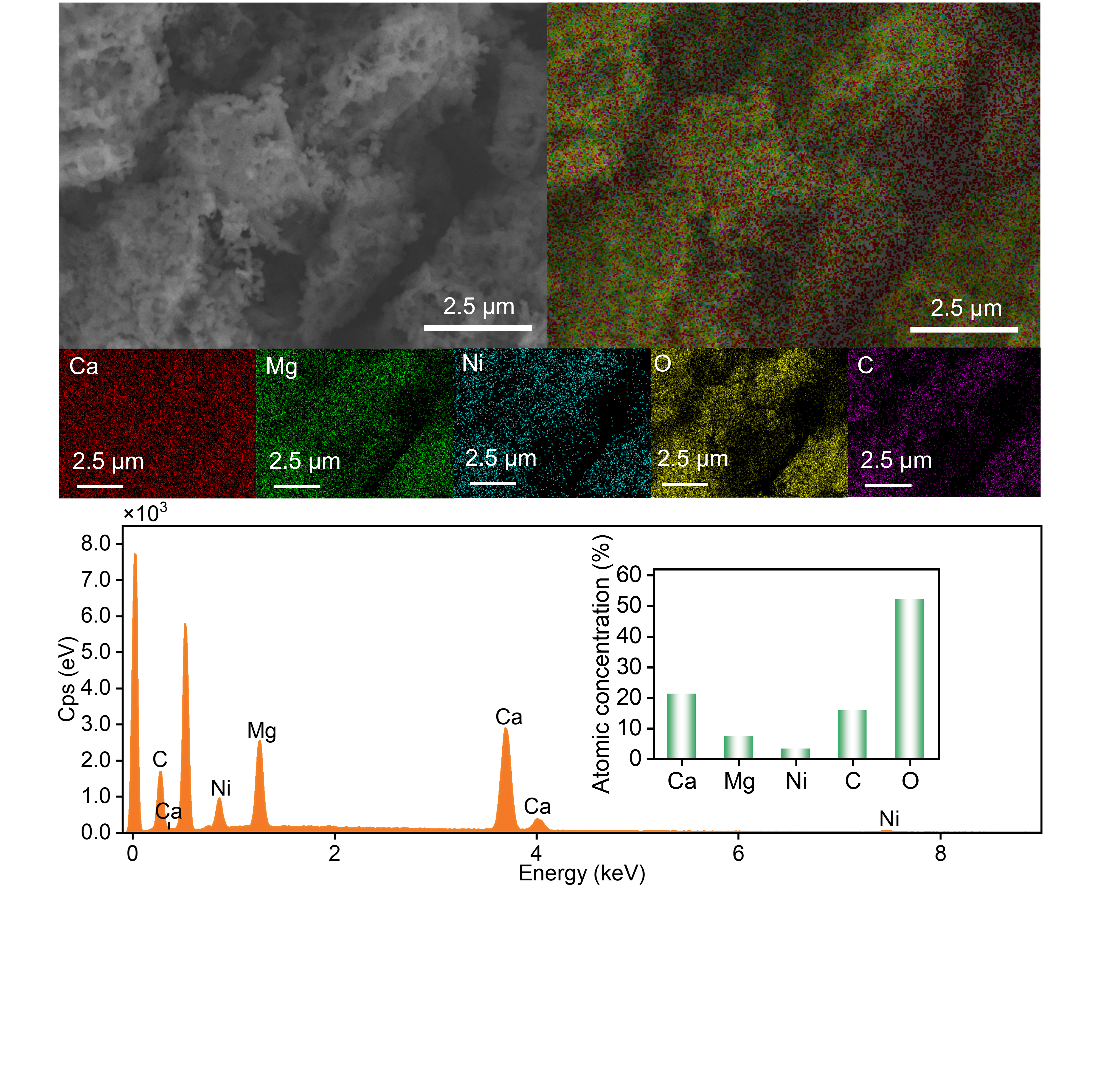


**Figure S25.** SEM-EDS analysis of Ni2 8.7 after the 1^st^ CO_2_ capture step.


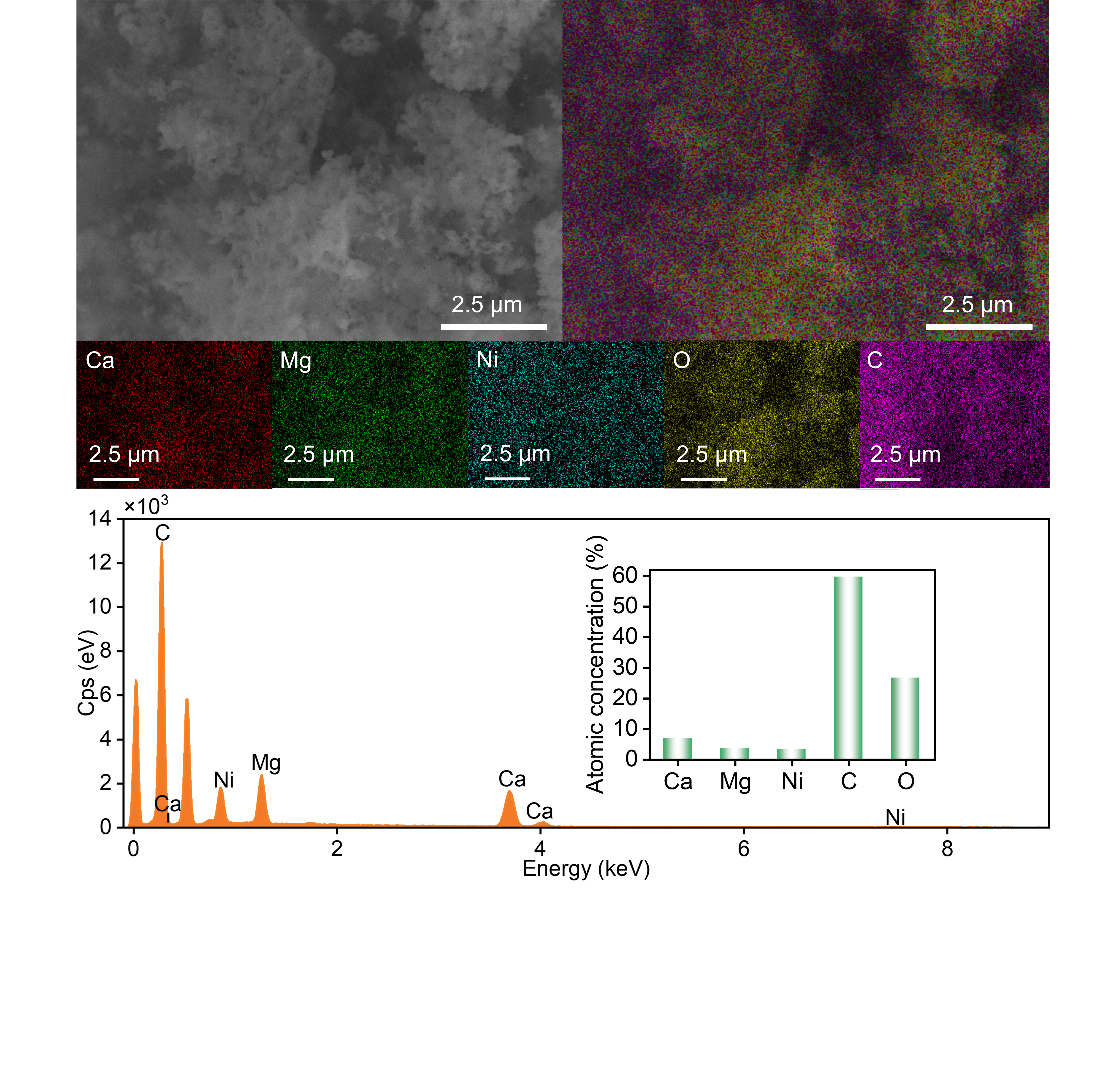


**Figure S26.** SEM-EDS analysis of Ni2 8.7 after the CO_2_ conversion step.


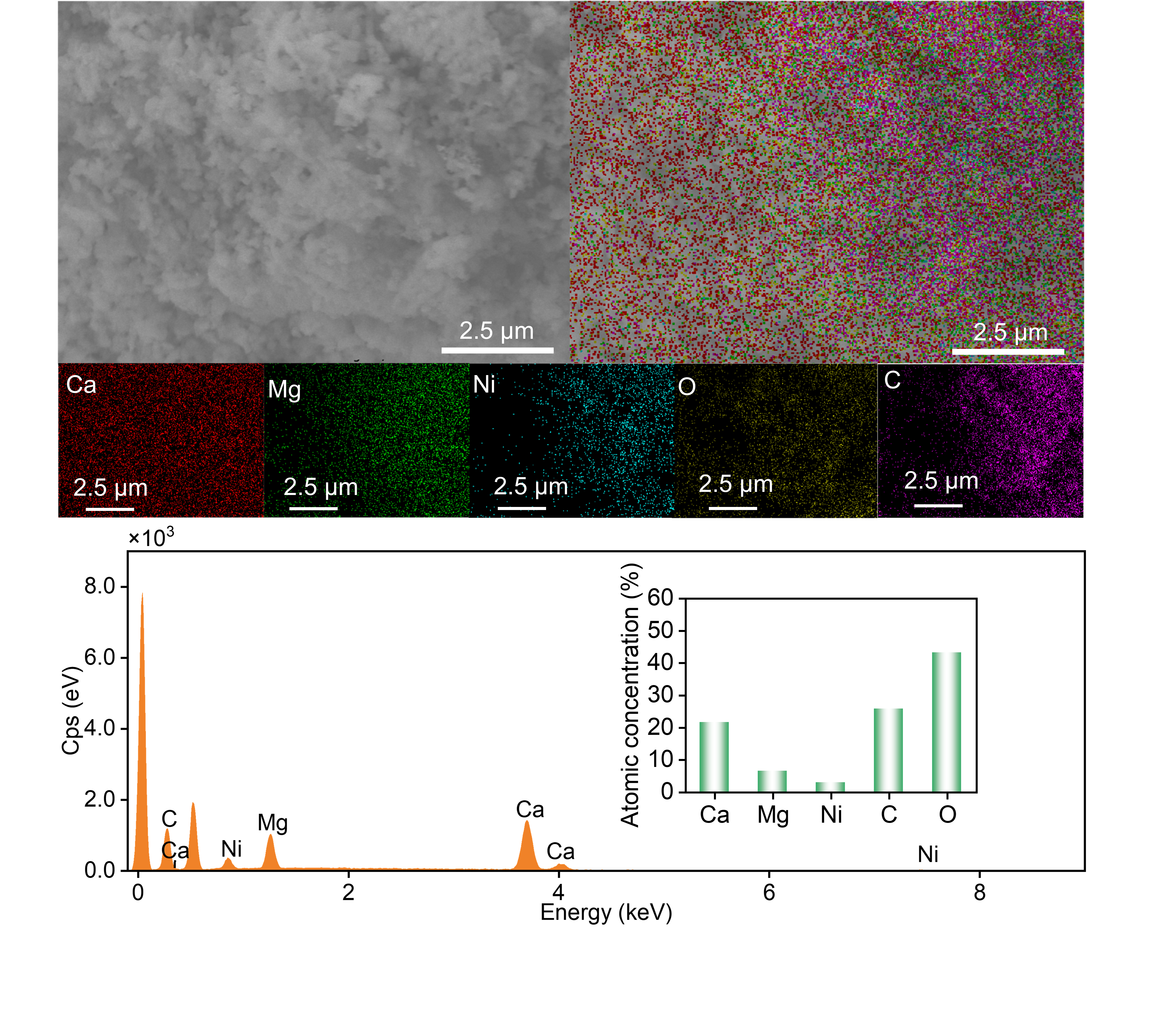


**Figure S27.** SEM-EDS analysis of Ni2 8.7 after the 2^nd^ CO_2_ capture step.

**Figure S28.** XRD patterns of the spent Ni2 8.7 and Ni1 14.6 after 65 cycles of CaLDRM.

**Supplementary Tables**

**Table S1.** The actual content of different components in the as-prepared DFMs.

| DFMs | Actual content (wt.%)^[a]^ | | | CaO (wt.%)^[b]^ | Ca:Mg (mol:mol)^[b]^ |
| --- | --- | --- | --- | --- | --- |
|  | Mg | Ca | Ni |  |  |
| Ni2 4.8 | 10.85 | 37.50 | 4.81 | 52.60 | 2.08 |
| Ni2 8.7 | 8.76 | 31.76 | 8.72 | 44.44 | 2.20 |
| Ni2 13.4 | 8.55 | 30.63 | 13.40 | 42.86 | 2.17 |
| Ni2 17.2 | 7.66 | 27.33 | 17.21 | 38.24 | 2.16 |
| Ni1 14.6 | 8.53 | 30.92 | 14.58 | 43.26 | 2.20 |

[a] Determined by ICP-OES. [b] Calculated from the actual content of Ca and Mg.

**Table S2.** The amount of surface active sites and the Ni dispersion in the DFMs.

| DFMs | H_2_ adsorption capacity (mL)^[a]^ | Surface active sites (μmol)^[b]^ | Ni dispersion (%)^[c]^ |
| --- | --- | --- | --- |
| Ni2 4.8 | 0.0330 | 2.95 | 12.01 |
| Ni2 8.7 | 0.0975 | 8.71 | 19.59 |
| Ni2 13.4 | 0.1147 | 10.24 | 14.96 |
| Ni2 17.2 | 0.1005 | 8.97 | 10.21 |
| Ni1 14.6 | 0.0775 | 6.92 | 9.28 |

[a] Determined by H_2_ pulse chemisorption. [b] The available Ni atoms on the material surface, calculated based on that the H : Ni stoichiometry equals to 1. [c] Calculated from the molar amount of surface active sites and total Ni atoms in the measured sample.

**Table S3.** Specific surface area and pore volume of the materials in different states, determined by N_2_ physisorption.

| Materials | States | Specific surface area | Mesopores | | | | Macropores  (50 ~ 200 nm) | |
| --- | --- | --- | --- | --- | --- | --- | --- | --- |
|  |  |  | (2 ~ 10 nm) | | (10 ~ 50 nm) | |  |  |
|  |  | (m^2^ g^-1^) | Volume  (cm^3^ g^-1^) | Proportion  (%) | Volume  (cm^3^ g^-1^) | Proportion  (%) | Volume  (cm^3^ g^-1^) | Proportion  (%) |
| CaMg | As-prepared | 44.0 | 0.0257 | 22.60 | 0.0457 | 40.08 | 0.0387 | 33.98 |
| Ni2 4.8 |  | 26.4 | 0.0185 | 14.73 | 0.0647 | 51.40 | 0.0426 | 33.87 |
| Ni2 8.7 |  | 28.7 | 0.0207 | 15.86 | 0.0608 | 46.55 | 0.0491 | 37.59 |
| Ni2 13.4 |  | 18.3 | 0.0115 | 12.55 | 0.0439 | 47.90 | 0.0362 | 39.55 |
| Ni2 17.2 |  | 16.9 | 0.0100 | 11.19 | 0.0460 | 51.54 | 0.0333 | 37.27 |
| Ni1 14.6 |  | 20.1 | 0.0137 | 13.58 | 0.0538 | 53.52 | 0.0331 | 32.89 |
| CaMg | Reduced^[a]^ | 33.3 | 0.0282 | 24.03 | 0.0491 | 41.82 | 0.0401 | 34.15 |
| Ni2 4.8 |  | 26.2 | 0.0184 | 15.27 | 0.0549 | 45.49 | 0.0473 | 39.23 |
| Ni2 8.7 |  | 31.1 | 0.0174 | 10.46 | 0.0922 | 55.46 | 0.0567 | 34.07 |
| Ni2 13.4 |  | 32.5 | 0.0239 | 15.40 | 0.0755 | 48.74 | 0.0556 | 35.86 |
| Ni2 17.2 |  | 26.2 | 0.0168 | 12.58 | 0.0610 | 45.66 | 0.0558 | 41.76 |
| Ni1 14.6 |  | 35.8 | 0.0275 | 14.77 | 0.0947 | 50.82 | 0.0641 | 34.40 |

[a] Obtained by reducing the as-prepared materials in 5 vol% H_2_/Ar at 650 °C for 30 minutes.

**Table S4.** The fitting results of H_2_-TPR profiles of the DFMs.

| DFMs | Fitted peaks | Peak position (°C)^[a]^ | Proportion (%)^[b]^ |
| --- | --- | --- | --- |
| Ni2 4.8 | α | 358 | 1.5 |
|  | β_1_ | 435 | 15.6 |
|  | β_2_ | 520 | 27.9 |
|  | γ | 772 | 55.0 |
| Ni2 8.7 | α | 353 | 2.9 |
|  | β_1_ | 415 | 7.8 |
|  | β_2_ | 511 | 31.9 |
|  | γ | 751 | 57.4 |
| Ni2 13.4 | α | 351 | 10.5 |
|  | β_1_ | 414 | 32.1 |
|  | β_2_ | 481 | 31.1 |
|  | γ | 668 | 26.3 |
| Ni2 17.2 | α | 347 | 24.3 |
|  | β_1_ | 415 | 29.6 |
|  | β_2_ | 472 | 27.6 |
|  | γ | 665 | 18.4 |
| Ni1 14.6 | α | 316 | 46.7 |
|  | β_1_ | 395 | 14.8 |
|  | β_2_ | 470 | 19.7 |
|  | γ | 665 | 18.9 |

[a] Determined by performing Gaussian fitting on the TPR profiles; [b] Calculated from the fitted peak area.

**Table S5**. Ni crystallite size of the DFMs in different states.

| DFMs | State | Ni (nm)^[a]^ |
| --- | --- | --- |
| Ni2 4.8 | Reduced | na.^[b]^ |
| Ni2 8.7 |  | 11.72 ± 0.20 |
| Ni2 13.4 |  | 13.69 ± 0.01 |
| Ni2 17.2 |  | 15.56 ± 0.42 |
| Ni1 14.6 |  | 23.75 ± 0.68 |
| Ni2 4.8 | After 20-hour steady-state DRM | 7.89 ± 0.40 |
| Ni2 8.7 |  | 14.83 ± 0.41 |
| Ni2 13.4 |  | 20.67 ± 0.76 |
| Ni2 17.2 |  | 21.27 ± 0.81 |
| Ni1 14.6 |  | 30.18 ± 0.05 |
| Ni2 8.7 | After 65 cycles of CaLDRM | 17.51 ± 0.55 |
| Ni1 14.6 |  | 32.06 ± 0.01 |

[a] Calculated from the characteristic diffraction peaks (2θ = 44.60° and 51.98°) in XRD patterns (Figures S7, S16 and S28) based on the Scherrer’s equation. [b] Not available, because the metallic Ni phase was unobserved in the XRD pattern of the reduced Ni2 4.8.

**Table S6.** CaO crystallite size of the materials in different states.

| Materials | State | CaO (nm)^[a]^ |
| --- | --- | --- |
| CaMg | Reduced | 21.04 ± 0.14 |
| Ni2 4.8 |  | 21.83 ± 0.49 |
| Ni2 8.7 |  | 20.84 ± 0.09 |
| Ni2 13.4 |  | 19.04 ± 0.52 |
| Ni2 17.2 |  | 19.11 ± 0.64 |
| Ni1 14.6 |  | 20.67 ± 0.97 |
| CaMg | Before 15 cycles of isothermal CO_2_ adsorption-desorption | 18.92 ± 0.57 |
| Ni2 4.8 |  | 19.24 ± 0.58 |
| Ni2 8.7 |  | 19.04 ± 0.71 |
| Ni2 13.4 |  | 18.87 ± 0.88 |
| Ni2 17.2 |  | 19.79 ± 0.27 |
| Ni1 14.6 |  | 18.61 ± 0.78 |
| CaMg | After 15 cycles of isothermal CO_2_ adsorption-desorption | 20.08 ± 0.17 |
| Ni2 4.8 |  | 19.62 ± 0.48 |
| Ni2 8.7 |  | 21.45 ± 0.53 |
| Ni2 13.4 |  | 20.79 ± 0.88 |
| Ni2 17.2 |  | 20.30 ± 0.69 |
| Ni1 14.6 |  | 18.61 ± 0.78 |

[a] Calculated from the characteristic diffraction peaks (2θ = 32.19° and 53.84°) in XRD patterns (Figures S7 and S11) based on the Scherrer’s equation.

**Table S7.** Comparison of the activity and stability of CaO-Ni based DFMs in the literature and this work.

| Sources | Materials | Temperature (°C) | CO_2_ uptake  (mmol_CO2_ g_material_^-1^)^[a]^ | CO_2_ in-situ conversion (%)^[a]^ | Number of cycles |
| --- | --- | --- | --- | --- | --- |
| **This work** | **Ni2 8.7** | **620** | **9.5** | **90** | **65** |
| References | 0.05PdNi/CaAl | 600 | 7.0 | 98 | 10 |
|  | CaO-0.3Ni/0.2CeO_2_ | 620 | 7.5 | 90 | 10 |
|  | Ni/Ca_85_Ce_15_ | 650 | 8.9 | 76 | 9 |
|  | Ni/NiCa | 650 | 9.1 | 68 | 10 |
|  | Ni-CaO-5(8.2) | 650 | 10.9 | 96 | 10 |
|  | Ca_60_Ni_10_Zr_30_ | 650 | 6.6 | 78 | 20 |
|  | Ni/(Ca@Si) | 650 | 3.9 | 89 | 10 |
|  | Ca_2_Ni_0.05_Ti_0.95_ | 700 | 6.4 | 44 | 30 |
|  | Ni/MgO-Al_2_O_3_ + CaO | 720 | 9.1 | 95 | 10 |
|  | NiCe/Ca@Zr | 720 | 5.9 | 44 | 25 |
|  | Ni-Ca_10_Mg | 720 | 11.4 | 65 | 10 |
|  | 10Ni-CaO-Ca_5_Al_6_O_14_ | 720 | 10.0 | 75 | 20 |
|  | Ni-CaMg | 720 | 7.9 | 73 | 10 |

[a] the CO_2_ uptake and in-situ conversion presented are the values in the last cycle.

**Reversible Reconstruction Kinetics Study**

Here, the experiments were implemented in the same reactor system under the same conditions as the performance evaluation of CaLDRM cycles. More details can be found in Experimental section. For kinetics study, 50 mg of the $\mathrm{Ni}_{8.7}^{2}$ DFM was used without inert quartz dilution. To assist the selection of the samples with different carbonation or decarbonation levels, a complete CaLDRM cycle was performed, consisting of 13-minute CO_2_ capture step, 21-minute CO_2_ conversion step and 2-minute Ar purging in between. The carbonation (car.) degree (%) and rate (% min^-1^) were estimated from the inlet and outlet gas flow rates during the CO_2_ capture step, based on the following equations:

$Car. degree\left( t \right)=\frac{\int_{0}^{t} \left[ F_{\mathrm{CO}_{2},in}(t)-F_{\mathrm{CO}_{2},out}(t) \right]\mathrm{dt}}{\int_{0}^{13} \left[ F_{\mathrm{CO}_{2},in}(t)-F_{\mathrm{CO}_{2},out}(t) \right]\mathrm{dt}}\times100$ (12)

$Car. rate(t)=\frac{d\left[ Car. degree(t) \right]}{\mathrm{dt}}$ (13)

where $Car. degree\left( t \right)$ and $Car. rate(t)$ are the carbonation degree (%) and carbonation rate (% min^-1^) at t minutes, respectively. $F_{\mathrm{CO}_{2},in}\left( t \right)$ and $F_{\mathrm{CO}_{2},out}\left( t \right)$ represent the inlet and outlet CO_2_ flow rates (ml min^-1^), respectively.

The decarbonation (dec.) degree (%) and rate (% min^-1^) were estimated from the inlet and outlet gas flow rates during the CO_2_ conversion step, based on the following equations:

$Dec. degree\left( t \right)=\frac{\int_{0}^{t} \left[ {F_{\mathrm{CO}_{2},out}\left( t \right)+F}_{\mathrm{CH}_{4},in}(t)-F_{\mathrm{CH}_{4},out}(t)+0.5F_{CO,out}(t)-0.5F_{H_{2},out}(t) \right]\mathrm{dt}}{\int_{0}^{21} \left[ {F_{\mathrm{CO}_{2},out}\left( t \right)+F}_{\mathrm{CH}_{4},in}(t)-F_{\mathrm{CH}_{4},out}(t)+0.5F_{CO,out}(t)-0.5F_{H_{2},out}(t) \right]\mathrm{dt}}\times100$ (14)

$Dec. rate(t)=\frac{d\left[ Car.degree (t) \right]}{\mathrm{dt}}$ (15)

where $Dec. degree\left( t \right)$ and $Dec. rate(t)$ are the decarbonation degree (%) and decarbonation rate (% min^-1^) at t minutes, respectively. $F_{\mathrm{CH}_{4},in}\left( t \right)$ represents the inlet flow rate (ml min^-1^) of CH_4_, and $F_{\mathrm{CO}_{2},out}\left( t \right)$, $F_{\mathrm{CH}_{4},out}\left( t \right)$, $F_{CO,out}\left( t \right)$ and $F_{H_{2},out}(t)$ represent the outlet flow rate (ml min^-1^) of CO_2_, CH_4_, CO and H_2_, respectively.

Based on the estimated carbonation and decarbonation degrees, we selected 7 samples (labeled as No. 1 ~ No. 7) to perform the kinetics study. Sample No. 1 ~ No. 4 were obtained from the CO_2_ capture step at the reaction stages of 0, 3, 7, and 13 minutes with carbonation degrees of 0, 40, 80 and 100%, respectively; Sample No. 5 ~ No. 7 were obtained from the subsequent CO_2_ conversion step at the reaction stages of 5, 12, and 21 minutes with decarbonation degrees of 40, 80 and 100%, respectively. Note that sample No. 1 was the reduced $\mathrm{Ni}_{8.7}^{2}$ DFM, and sample No. 4 was collected at the end of the CO_2_ capture step, which was the starting sample for the subsequent CO_2_ conversion step as well.

The phase transformation of the $\mathrm{Ni}_{8.7}^{2}$ DFM during the CaLDRM process was determined by the XRD patterns of the samples No. 1 ~ No. 7. The phase transformation rates were estimated by the variation of relative intensities between CaO and CaCO_3_ as well as MgO and Mg_x_Ni_1-x_O phases in the samples at different reaction stages, since the relative intensity of reversible phases is positively correlated with their relative content.^[9-10]^ The calculation formula is provided as follows:^[10]^

$I[i/j]=\frac{A_{i}}{A_{j}}$  (16)

where $I[i/j]$ represents the relative intensity of phase i to phase j (i, j can be one of the CaO, CaCO_3_, MgO and Mg_x_Ni_1-x_O), and $A_{i}$and $A_{j}$ are the fitted area of the characteristic diffraction peak of phase i and phase j, respectively.

The phase migration of the $\mathrm{Ni}_{8.7}^{2}$ DFM during the CaLDRM process was analyzed by performing SEM-EDS elemental mapping for the samples No. 1 ~ No. 7. The phase migration rates were estimated by the variation of surface atomic ratios of (Mg+Ni) and Ca as well as Ni and Mg in the samples at different reaction stages. The calculation formula is shown as follows:

$SA[i/j]=\frac{at(i)}{at(j)}$ (17)

where $SA[i/j]$ represents the surface atomic ratio of element i to element j (i, j can be one of the Ni, Mg, (Mg+Ni) and Ca), and $\mathrm{at}(i)$ and $\mathrm{at}(j)$ are surface atomic concentration (%) of element i and element j, respectively.


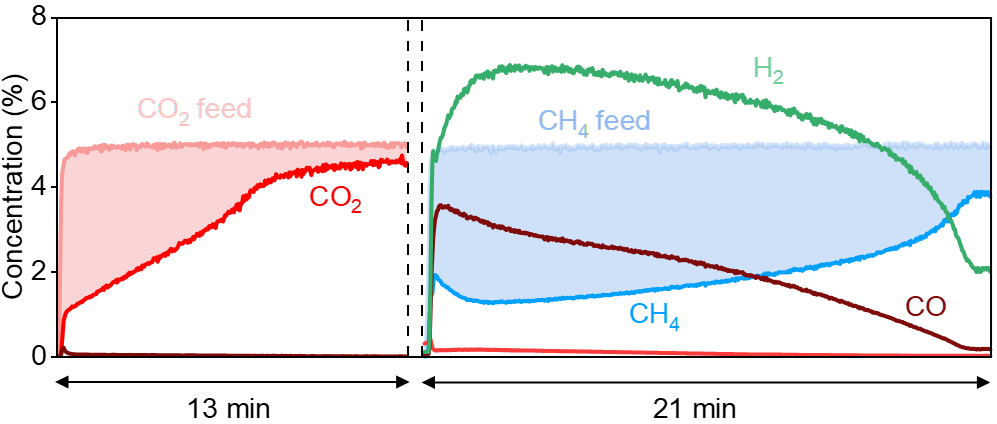


**Figure S29.** The concentrations of the inlet (feed) and outlet (product) gases during CaLDRM implemented on the $\mathrm{Ni}_{8.7}^{2}$ DFM at 620°C, where the shadow regions indicate the consumption of CO_2_ or CH_4_; 50 mg of the material without inert quartz dilution was used for tests, which was firstly reduced in 5 vol.% H_2_/Ar at 650°C (heating rate: 10°C min^-1^) for 30 minutes before starting CaLDRM process; one cycle comprises 13-minute CO_2_ capture step (in 5 vol.% CO_2_/Ar), 21-minute CO_2_ conversion step (in 5 vol.% CH_4_/Ar) and 2-minute Ar purging in between.


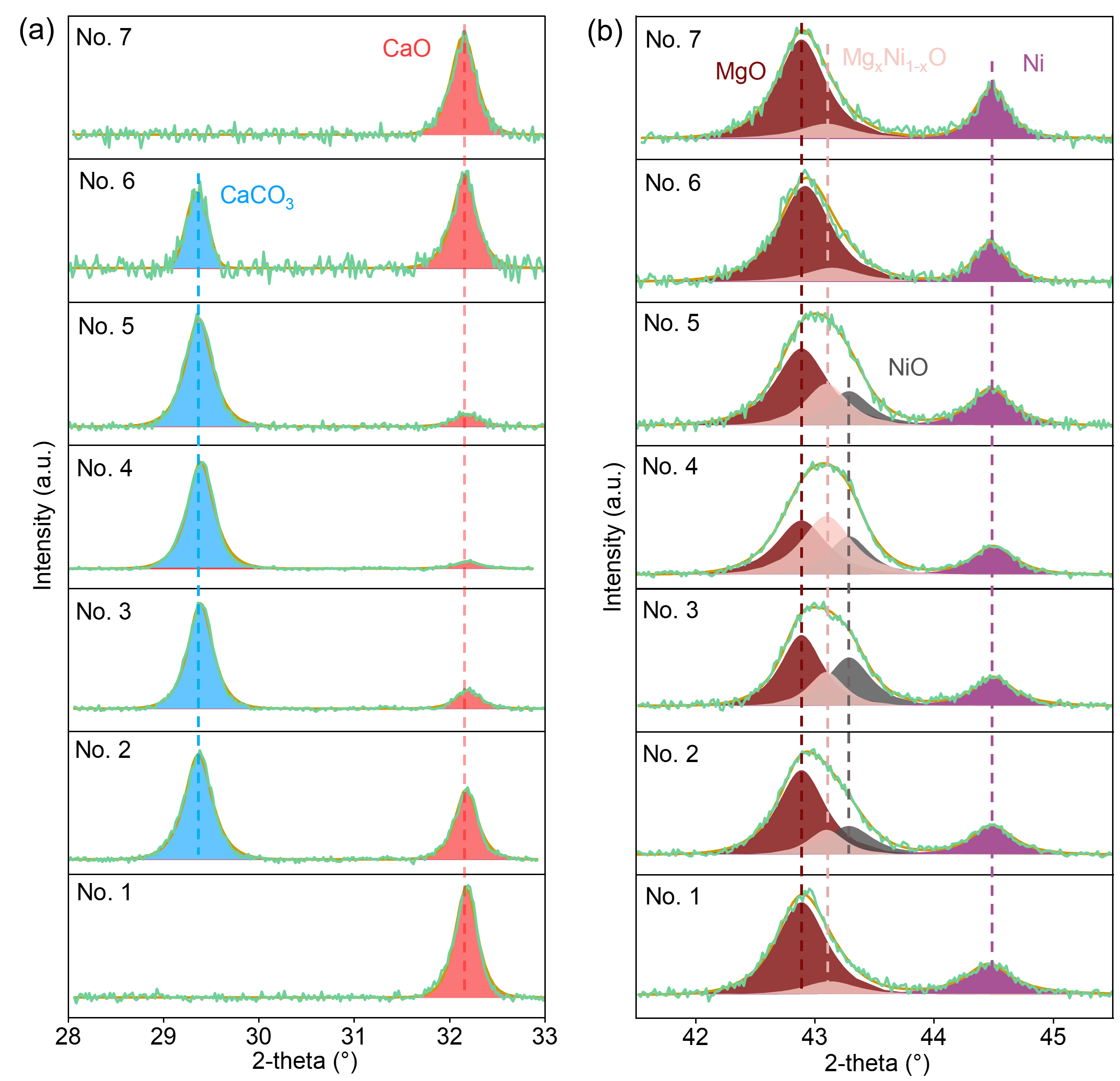


**Figure S30.** XRD patterns and the fitting on the characteristic peaks, within the region of 28° ~ 33° (a) and 42° ~ 45° (b), for the $\mathrm{Ni}_{8.7}^{2}$ samples at progressive carbonation and decarbonation levels; Sample No. 1 ~ No. 4 were collected from the CO_2_ capture step at the reaction stages of 0, 3, 7, and 13 minutes with carbonation degrees of 0, 40, 80 and 100%, respectively; Sample No. 5 ~ No. 7 were collected from the subsequent CO_2_ conversion step at the reaction stages of 5, 12, and 21 minutes with decarbonation degrees of 40, 80 and 100%, respectively.

**Discussion**: In sample No. 1 (the reduced $\mathrm{Ni}_{8.7}^{2}$), neither CaCO_3_ nor NiO phases are detected, indicating that the material initially provides sufficient CaO and metallic Ni as active phases. During the CO_2_ capture step (samples No. 1 to No. 4), the CaO diffraction peak gradually weakens while the CaCO_3_ peak intensifies, confirming the transformation of CaO into CaCO_3_. Concurrently, a NiO phase emerges; its intensity first rises and then declines (samples No. 2 to No. 4). Meanwhile, the Mg_x_Ni_1-x_O peak progressively strengthens as the MgO peak diminishes, indicating that metallic Ni is oxidized to NiO, which subsequently incorporates into MgO to form the solid solution. During the subsequent CO_2_ conversion step (samples No. 4 to No. 7), the CaCO_3_ peak gradually decreases while the CaO peak increases, eventually recovering to the initial state (compare sample No. 7 with sample No. 1), demonstrating complete decomposition of CaCO_3_ to CaO. At the same time, the NiO phase disappears, and the Mg_x_Ni_1-x_O peak weakens, accompanied by the restoration of MgO and metallic Ni intensities to their original levels (compare sample No. 7 with sample No. 1). This evolution confirms the effective exsolution of metallic Ni from the solid solution upon reduction. Collectively, these observations provide clear evidence for the dynamic, reversible phase transformation of the material during the CaLDRM process.


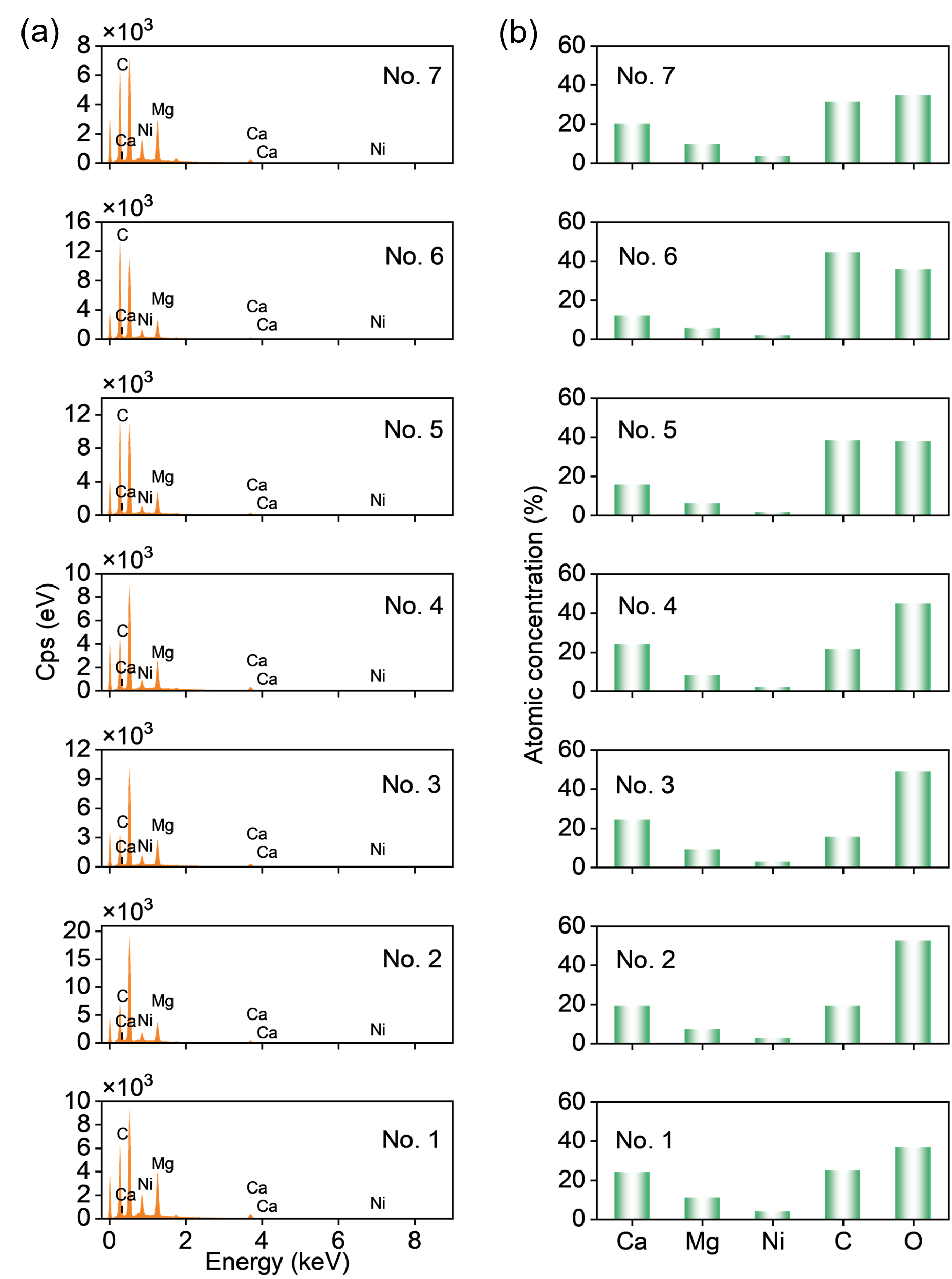


**Figure S31.** (a) SEM-EDX spectra and (b) the surface atomic concentrations for the samples No. 1 ~ No. 7.

# Supplementary References

[1] J. Hu, Y. Jiang, Q. Gao, Y. Zhao, S. Dai, X. Li, W. Wei, *Chem. Eng. J.* **2025**, *505*, 159237.

[2] U. Holzwarth, N. Gibson, *Nat. Nanotechnol.* **2011**, *6*, 534.

[3] C. H. Bartholomew, R. B. Pannell, *J. Catal.* **1980**, *65*, 390-401.

[4] S. Ewald, S. Standl, O. Hinrichsen, *Appl. Catal. A* **2018**, *549*, 93-101.

[5] M. Lindblad, L. P. Lindfors, T. Suntola, *Catal. Lett.* **1994**, *27*, 323-336.

[6] S. Das, A. Jangam, Y. Du, K. Hidajat, S. Kawi, *Chem. Commun.* **2019**, *55*, 6074-6077.

[7] R. Han, S. Xing, Y. Wang, L. Wei, Z. Li, C. Yang, C. Song, Q. Liu, *Sep. Purif. Technol.* **2023**, *307*, 122808.

[8] J. Cai, Y. Han, S. Chen, E. J. Crumlin, B. Yang, Y. Li, Z. Liu, *J. Phys. Chem. C* **2019**, *123*, 12176-12182.

[9] L. Alexander, H. P. Klug, *Anal. Chem.* **1948**, *20*, 886-889.

[10] H. Toraya, *J. Appl. Crystallogr.* **2016**, *49*, 1508-1516.
